# Development of *Klebsiella pneumoniae* Capsule Polysaccharide-Conjugated Vaccine Candidates Using Phage Depolymerases

**DOI:** 10.3389/fimmu.2022.843183

**Published:** 2022-03-21

**Authors:** Tzu-Lung Lin, Feng-Ling Yang, Chien-Tai Ren, Yi-Jiun Pan, Kuo-Shiang Liao, I-Fan Tu, Yu-Pei Chang, Yang-Yu Cheng, Chung-Yi Wu, Shih-Hsiung Wu, Jin-Town Wang

**Affiliations:** ^1^ Department of Microbiology, National Taiwan University College of Medicine, Taipei, Taiwan; ^2^ Institute of Biological Chemistry, Academia Sinica, Taipei, Taiwan; ^3^ Genomics Research Center, Academia Sinica, Taipei, Taiwan; ^4^ Department of Microbiology, School of Medicine, China Medical University, Taichung, Taiwan; ^5^ Department of Internal Medicine, National Taiwan University Hospital, Taipei, Taiwan

**Keywords:** capsule polysaccharide, depolymerases, *Klebsiella pneumoniae*, CPS-conjugated vaccines, bactericidal.

## Abstract

*Klebsiella pneumoniae* is an important pathogen associated with nosocomial infection and has developed increasing resistance to antibiotics such as extended-spectrum β-lactams and carbapenem. In recent years, *K. pneumoniae* isolates have emerged as a major cause of global community-acquired infections such as pneumonia and pyogenic liver abscess. Although serotypes K1 and K2 have been identified as the predominant capsular types associated with invasive infections, no *K. pneumoniae* vaccine is commercially available, probably due to immunogenicity loss in the traditional depolymerization method to obtain capsule polysaccharide (CPS) for the preparation of conjugated vaccine. In this study, we successfully retained immunogenicity by using K1 (K1-ORF34) and K2 (K2-ORF16) CPS depolymerases that were identified from phages to cleave K1 and K2 CPSs into intact structural units of oligosaccharides with intact modifications. The obtained K1 and K2 oligosaccharides were separately conjugated with CRM197 carrier protein to generate CPS-conjugated vaccines. Immunization experiments of mice showed both K1 and K2 CPS-conjugated vaccines induced anti-CPS antibodies with 128-fold and 64-fold increases of bactericidal activities, respectively, compare to mice without vaccinations. Challenge tests indicated that K1 or K2 CPS-conjugated vaccine and divalent vaccine (a mixture of K1 and K2 CPS-conjugated vaccines) protected mice from subsequent infection of *K. pneumoniae* by the respective capsular type. Thus, we demonstrated K1 and K2 CPS-conjugated vaccines prepared by CPS depolymerases is a promising candidate for developing vaccines against human *K. pneumoniae* infections.

## Introduction

*Klebsiella pneumoniae* is a pathogen that causes various community-acquired and hospital-acquired diseases, including pneumonia, sepsis, and urinary tract infection (1). Moreover, *K. pneumoniae* is responsible for approximately 10% of nosocomial infections and increasingly exhibits multiple drug resistance to antibiotics, such as extended-spectrum beta-lactamases (ESBLs) and carbapenemases (2). In recent years, the “hyper-virulent” *K. pneumoniae* strains have emerged as a clinically relevant pathogen worldwide, infecting healthy people and causing serious disseminated infections (3–5).

Community-acquired pyogenic liver abscess (PLA) caused by *K. pneumoniae* has been reported increasingly worldwide, especially in Asia (6–15). Moreover, *K. pneumoniae* has been reported to cause invasive infections leading to organ abscesses (such as kidney, spleen, brain, and prostate abscesses), necrotizing fasciitis, and severe pneumonia with bacteremia (5, 16, 17). The most common capsular type of the strains that cause PLA is K1 followed by K2 (8, 18, 19). Recent studies demonstrated that K1 and K2 strains also caused other invasive infections such as necrotizing fasciitis and bacteremic community-acquired pneumonia (16, 17, 20).

To battle infections caused by these encapsulated pathogens, bacterial capsule-targeted vaccines, such as those for *Streptococcus pneumoniae*, *Neisseria meningitides*, and *Haemophilus influenzae*, have achieved considerable success (21). Although the design of *K. pneumoniae* K1 capsule polysaccharide (CPS) vaccine was first reported in 1985 (22) and the designs of polyvalent *K. pneumoniae* CPS vaccines were then reported in 1986 (six-valent) and 1988 (24-valent) (23, 24), there is still no clinical *K. pneumoniae* vaccine available. Previous studies demonstrated that the polysaccharide vaccine only induced T-cell-independent immunity, failing to elicit immunological memory or promote production of high-affinity antibodies (25). However, a *K. pneumoniae* CPS-protein-conjugated vaccine is expected to be more effective against infections caused by this bacterium.

**Figure 1:**
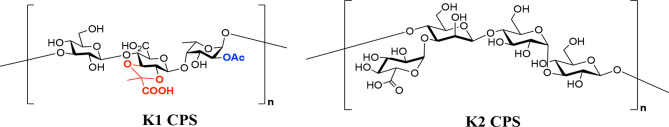
Chemical structures of capsular polysaccharides (CPS) of K1 and K2.

K1 and K2 CPSs are extended polymeric molecules composed of multiple repeat units of sugars (26) (**Figure 1**); thus, their large molecular weights and sticky feature make them difficult to form conjugated vaccines. Although depolymerization of CPS can increase the efficiency of protein conjugation, chemical reagents used to reduce CPS units (such as trifluoroacetic acid, ammonium hydroxide, and acetic acid) cause the loss of CPS modification (acetylation or pyruvation) and impair the induction of immune responses upon vaccination (26). In this study, we successfully used specific K1 and K2 types of *K. pneumoniae* CPS depolymerases cloned from specific phages to obtain the depolymerized CPS without losing their important immunogenic modifications; then, the depolymerized CPS was used to synthesize capsule-conjugated vaccine candidates for the K1 and K2 types of *K. pneumoniae*, which was evaluated favorably in a mouse model.

## Results

### Isolation of Phage That Infects *K. pneumoniae* 1611E Strain (capsular type K2)

Our previous study identified a K1 capsule depolymerase (K1-ORF34) from NTUH-K2044-K1-1 **(**a K1-specific phage; accession number AB716666) (27). In order to isolate a K2-capsule-degrading enzyme, phage capable of infecting the *K. pneumoniae* 1611E strain (capsular type K2) was isolated from untreated water as clear plaque with translucent halo (designated 1611E-K2-1). The sensitivity of capsular type K2 phage 1611E-K2-1 was evaluated against 7 other capsular-type-K2 strains, whose capsular types were determined by PCR using *wzy* primers. Results showed that phage 1611E-K2-1 could infect all capsular type K2 strains.

### Identification of the Putative K2 Capsule Depolymerase

**Figure 2:**
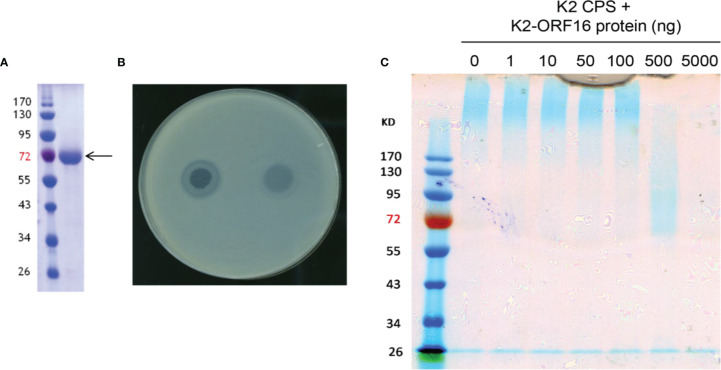
The enzymatic activity of purified K2-ORF16 protein. **(A)** The purified K2-ORF16 protein (right column; indicated with the arrow) was subjected to SDS-PAGE and stained with Coomassie brilliant blue; the sizes (in kDa) of protein marker bands are indicated next to the left column. **(B)** A clear spot surrounded by a translucent halo (left) and a translucent spot (right) were observed when the phage 1611E-K2-1 and purified K2-ORF16 protein (respectively) were spotted on a plate overlaid with top agar containing the *K pneumoniae* 1611E strain. **(C)** Extracted K2 CPS was treated with various amounts (0-5000 ng) of K2-ORF16 protein and subjected to SDS-PAGE followed by staining with Alcian blue. Left-most column contains protein markers (with sizes in kDa indicated to the left).

The full genome of phage 1611E-K2-1 was determined to be 47,797 bp in length **(**accession number MG197810). Annotation of the genome sequences predicted that this phage should contain 17 open reading frames (ORFs) of more than 500 bp. Our analysis on the ORFs of phage 1611E-K2-1 revealed that the predicted ORF16 exhibited 46% amino acid identity with a tailspike 63D sialidase, suggesting that the protein might correspond to a capsule depolymerase. Subsequently, the K2-ORF16 gene was cloned and expressed in *E. coli*, and the purity of the purified recombinant K2-ORF16 protein was verified (**Figure 2A**). Upon spotting a plate inoculated with top agar containing *K. pneumoniae* 1611E, the recombinant K2-ORF16 protein generated a translucent spot resembling the plaque halo (**Figure 2B**). To confirm the putative depolymerase activity of the purified protein, CPS was extracted from 1611E and then treated *in vitro* with K2-ORF16 protein; the resulting products were separated *via* SDS-PAGE and stained with Alcian blue (**Figure 2C**). The results showed that the K2-ORF16 protein degrades the K2 CPS. The specificity of this enzyme for capsular type K2 was further confirmed in 7 other capsular-type-K2 strains. These results demonstrated that the K2-ORF16 protein is a K2 capsule depolymerase.

### Analysis of K1-ORF34 Capsule Depolymerase-Digested CPS

To verify the CPS modifications remain intact, K1 CPS treated by K1-ORF34 capsule depolymerase was digested and further analyzed. After incubation with purified K1-ORF34 protein, K1 CPS was reduced to oligosaccharides. Mass spectrometry revealed that the majority of oligosaccharides were hexa-saccharide (m/z 1145), contained dimer of a structural trisaccharide repeating unit: (→3)-β-D-Glc-(1→4)-[2,3-(*S*)-pyruvate]-β-D-GlcA-(1→4)-α-L-Fuc-(1→), and had the unusual feature of extensive pyruvation of glucuronic acid and acetylation of fucose at C2-OH or C3-OH with nonasaccharide (trimer) and trisaccharide (monomer) as minor components (**Supplementary Figures 1A-C**). MS/MS analysis revealed the molecular weight of the oligosaccharide is 1145, suggesting that glucuronic acid was reduced to a double-bond derivative that absorbed wavelength of 232 nm. The major enzymatic product is a hexasaccharide **1**: D-GlcA*-(1→4)-α-L-Fuc-(1→3)-β-D-Glc-(1→4)-[2,3-(S)- pyruvate]-β-D-GlcA-(1→4)-α-L-Fuc-(1→3)-β-D-Glc; GlcA*: 4,5-unsaturated glucuronic acid (delta 4,5 GlcA). NMR spectra (δ 1.6, s and δ 1.55, s for CH_3_; δ 2.1, s and δ 2.05, s for OCOCH_3_) indicated that the pyruvate and acetyl modifications were still present (**Supplementary 1D**). These results suggested that the K1-ORF34 enzyme belongs to the lyase class of enzymes and can be used in the preparation of K1 carbohydrate antigens for the development of K1 CPS-conjugated vaccine.

### Analysis of K2-ORF16 Capsule Depolymerase-Digested CPS

To verify the CPS modifications remain intact, K2 CPS treated by K2-ORF16 capsule depolymerase was digested and further analyzed. The spectra showed that the major components of K2-ORF16-digested CPS were tetrasaccharide monomer **2** (m/z 703, Man-[(3←1)-α-GlcA]-(1→4)-α-Glc-(1→3)-β-Glc) and the minor components were dimer (octasaccharide) and trimer (dodecasaccharide) (**Supplementary Figures 2A–C**). The product with m/z of 703 was further examined by ESI-MS/MS analysis. The spectrum showed that this product contained three hexoses and one uronic acid, consistent with the previously reported structure of K2 CPS (28). We had assigned the NMR spectra and compared the reported K2 CPS spectra (28). Based on the information, the ^13^C chemical shift at C-4 of mannose was assigned as δC 73.3. In our NMR studies, the up-field 13C chemical shift at the same position of the oligosaccharide was found to be δC 66.1, suggesting the non-reducing end was located at C-4 position of mannose (**Supplementary Figures 2D–F** and **Supplementary Table 1**). These results indicated that the K2-ORF16 enzyme belongs to a kind of hydrolase and can apply for the preparation of K2 carbohydrate antigens for the development of K2 CPS-conjugated vaccine.

### K1 and K2 CPS-Conjugated Vaccines

**Scheme 1:**
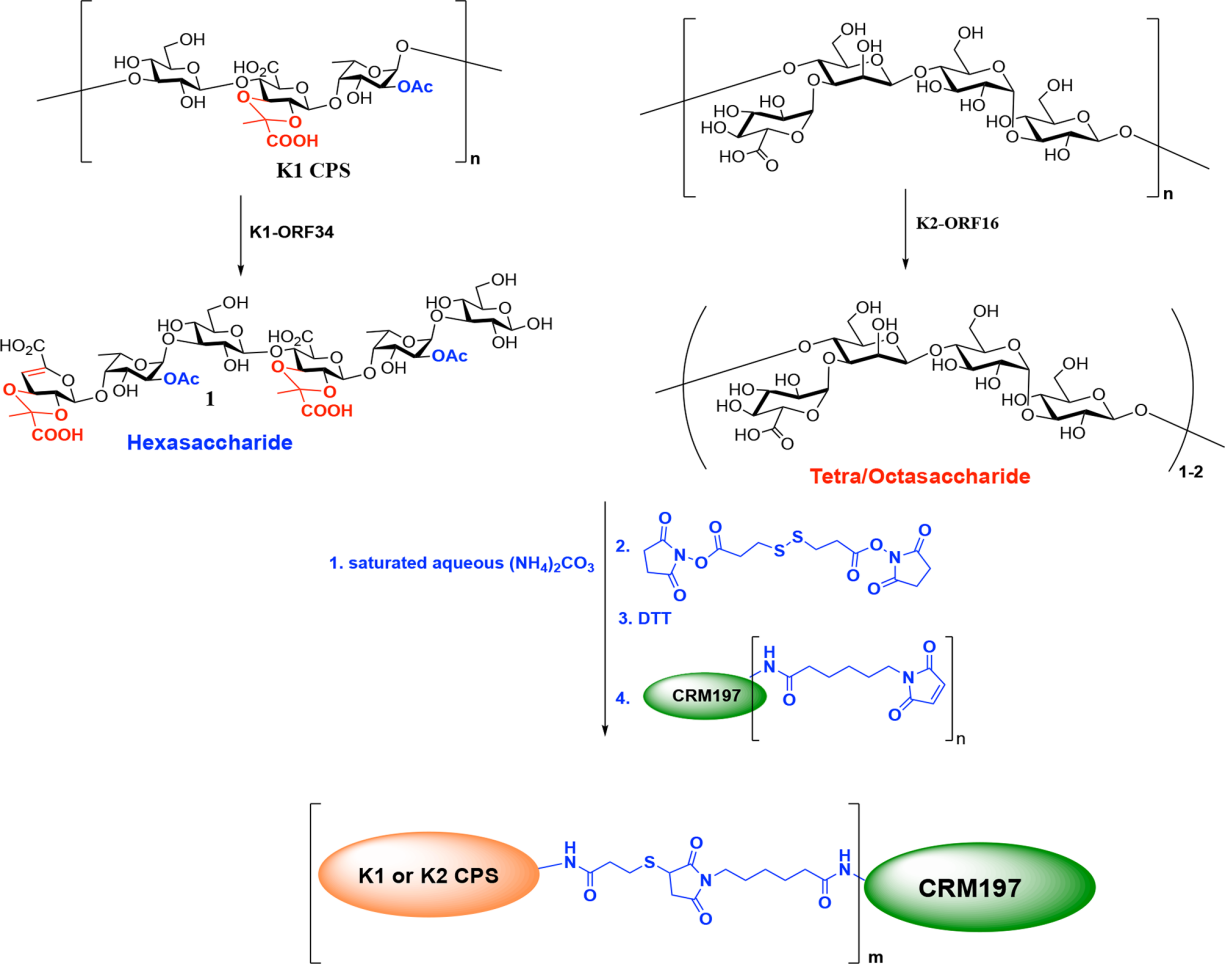
Preparation of K1 or K2 CPS conjugated Vaccines.

The MS analysis showed that the digested K1 and K2 oligosaccharides used for conjugation consisted primarily of hexasaccharides **1** (two repeat units of K1 CPS) and tetrasaccharides/octasaccharides **2** (one/two repeat unit of K2 CPS), respectively. The compounds **1** and **2** were subjected to the Kochetkov amination using ammonium carbonate to change the anomeric hydroxyl group to the amine group; then, the glycosyl amines were reacted with DTSSP linker to form the glycosyl disulfides. After DTT treatment, the K1/K2 digested CPS-SH was reacted with *CRM197-maleimid* to form the K1 and K2 CPS-conjugated vaccine candidates (**Scheme 1**). The epitope ratio (K1 = 5.38, K2 = 7.42, sugar/protein) was determined by MALDI-TOF mass spectrometry (**Supplementary Figures 3**, **4**).

### Toxicology Assay

**Figure 3:**
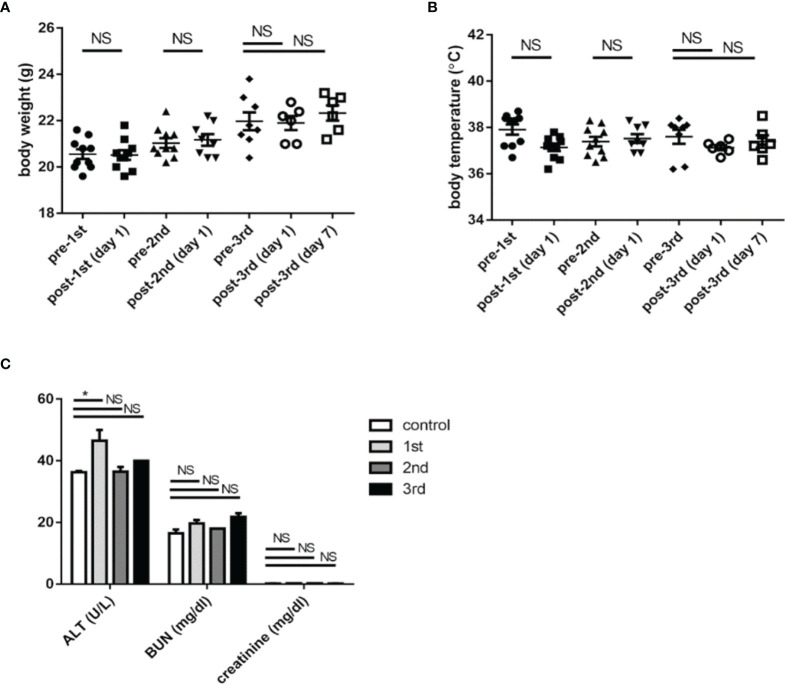
The safety of K1 CPS-conjugated vaccine. **(A)** The body weights of mice were recorded before and one day after each administration (1st, 2nd, and 3rd) of K1 CPS-conjugated vaccine. **(B)** The rectal temperatures of mice were recorded before and one day after each administration of K1 CPS conjugated vaccine. **(C)** Blood levels of markers of liver (ALT) and renal (BUN and creatinine) functions were determined one week after each administration of K1 CPS-conjugated vaccine. Note: body weight and temperature data of individual animals were plotted in panels A and B; the horizontal bar indicates the mean, and the error bar indicates SEM. For **(C)**, blood level values are plotted as mean + SEM. The data were analyzed using the Tukey’s multiple comparison *post hoc* one-way ANOVA analysis. *P < 0.05; NS, not significant. Although ALT levels are slightly higher 1 week after vaccination. *ALT titers are all within normal range. Similar observations were noted in K2 vaccinated mic but serum biochemical tests were not performed.

The body weight (**Figure 3A**) and rectal temperature measurements (**Figure 3B**) of mice were recorded right before and one day after each vaccination of K1 CPS-conjugated vaccine. Sera of mice were collected one week after the 1^st^, 2^nd^, and 3^rd^ administrations of K1 CPS-conjugated vaccine, and blood levels of liver (ALT) and renal (BUN and creatinine) markers were determined (**Figure 3C**). The mouse body weight measurements from before vaccination and one day after vaccination did not have significant difference; rectal temperature measurements for mice administered with K1 CPS-conjugated vaccine were all in the normal range. The blood level measurements of liver and renal markers were also all in the normal range and did not alter in vaccinated mice compared to control mice. These results suggested no side effects in mice administered with three doses of K1 CPS-conjugated vaccine. Observations were similar in K2 vaccinated mice, except no serum biochemical tests were done.

### Antibody Response and Serum Bactericidal Assay

**Figure 4:**
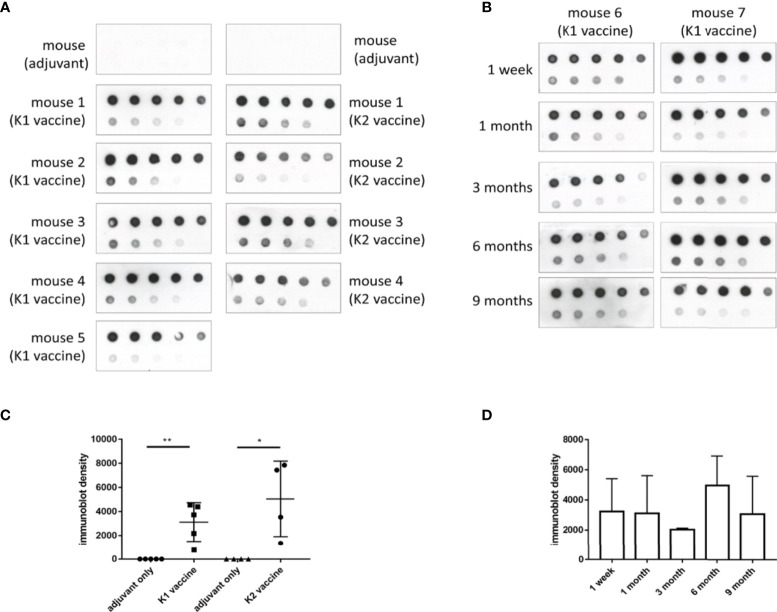
The induction and persistence of antibodies against CPS in mice administered with K1 or K2 CPS-conjugated vaccines. Purified K1 or K2 CPS was subjected to a serial two-fold dilution, and various amounts (2000 ng to 7.8125 ng, from the upper left to the lower right) were spotted to membranes. Membranes were then blotted with sera (1:1000 dilution) from mice that had been administered with adjuvant or K1 or K2 CPS-conjugated vaccine. **(A)** Dot blotting of sera collected one week after the 3rd vaccine injection. **(B)** Dot blotting of sera collected from either of the two K1 vaccinated mice at various intervals (1 week to 9 months) after the 3rd anti-K1 vaccine injection. **(C)** The density of dots spotted with 31.25 ng of CPS in **(A)** was quantified by using ImageJ software (Version 1.53k, USA). The antibodies against K1 or K2 CPS in mice administered with K1 or K2 CPS-conjugated vaccines were significantly higher than those received adjuvant only. The data were analyzed using the two-tailed unpaired t test. *P < 0.05; **P < 0.01. **(D)** The density of dots spotted with 31.25 ng of CPS in **(B)** was quantified by using ImageJ software. The antibodies against K1 CPS in mice administered with K1 CPS-conjugated vaccine at various intervals (1 week to 9 months) after the 3rd anti-K1 vaccine injection were not significantly different. The data were analyzed using the Tukey’s multiple comparison *post hoc* one-way ANOVA analysis.

Sera of mice that were administered with three doses of K1 or K2 CPS-conjugated vaccine by IM injection were collected for assessments of antibody production and bactericidal activity. The results showed antibodies induced by the K1 or K2 CPS-conjugated vaccine interacted with their original CPS (**Figure 4A, C**). The major induced antibody of K1 CPS-conjugated vaccine was IgG1 (**Supplementary Figure 5**). The persistence of antibodies was still observed 9 months after administration of the last vaccine booster dose (**Figure 4B, D**).

**Figure 5:**
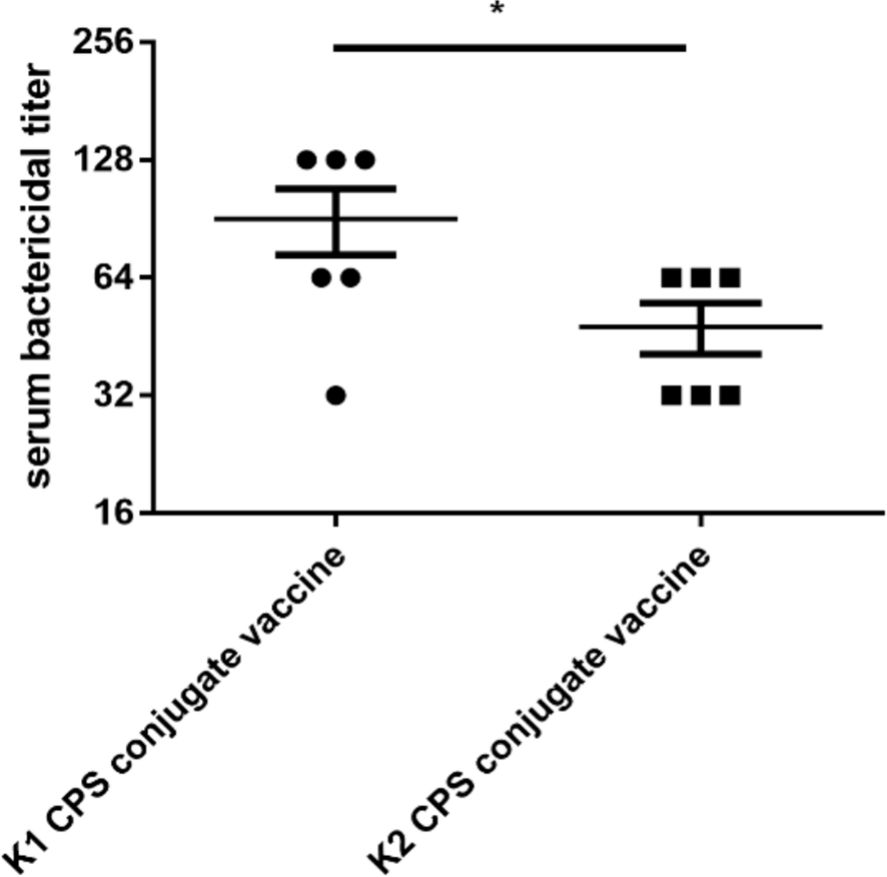
The bactericidal titers of serum from mice administered with K1 or K2 CPS-conjugated vaccines. The data were analyzed using the two-tailed unpaired t test. *P < 0.05.

The bactericidal activity assay results revealed that the K1 bacteria bactericidal titer of sera from mice immunized with K1 CPS-conjugated vaccine was 32~128, which means sera from mice immunized by K1 CPS-conjugated vaccine killed more than 50% of K1 bacteria at a dilution of up to 1: 128 (i.e., titer of 128). On the other hand, sera from non-immunized mice did not exhibit appreciable bactericidality even at the lowest (1: 2) tested dilution. For the K2 bacteria, the bactericidal titer of sera from mice immunized with K2 CPS-conjugated vaccine was 32~64 (**Figure 5**).

**Figure 6:**
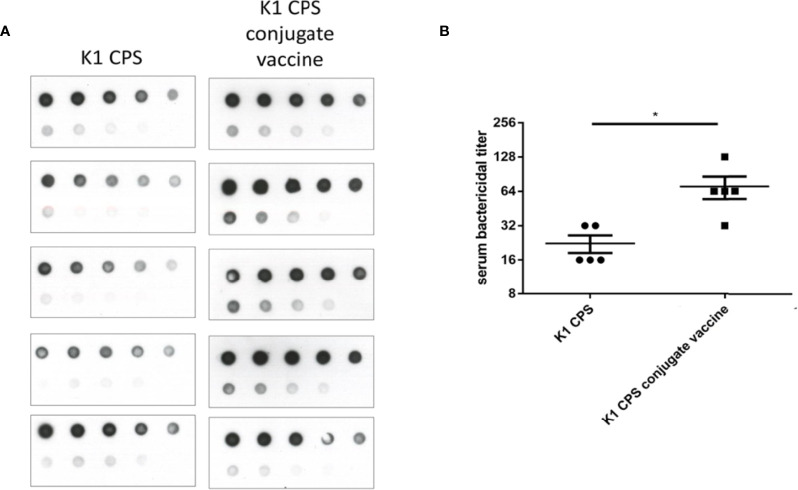
Comparisons of immunization with K1 or K1 CPS conjugated vaccine. The induction of anti-CPS antibodies and serum bactericidal activity in mice received K1 crude CPS (2 μg) or K1 CPS conjugated vaccine (2 μg) were compared. **(A)** The amounts of anti-K1 antibodies in mice received K1 CPS conjugated vaccine (determined by immunoblotting) were higher than those in mice received K1 crude CPS. **(B)** Accordingly, the serum bactericidal activity was also significantly higher in mice received K1 CPS conjugated vaccine. The data were analyzed using the two-tailed unpaired t test. *P < 0.05.

Notably, cross-capsular-type protection was not observed. Comparisons of immunization with K1 or K1 CPS conjugated vaccine. The serum bactericidal activities were higher in conjugated vaccines (**Figures 6A, B**). Therefore, we can conclude that the CPS-conjugated vaccines successfully induced production of capsular-type-specific antibody in mice with promising bactericidal activity.

### Protection Assay

**Figure 7:**
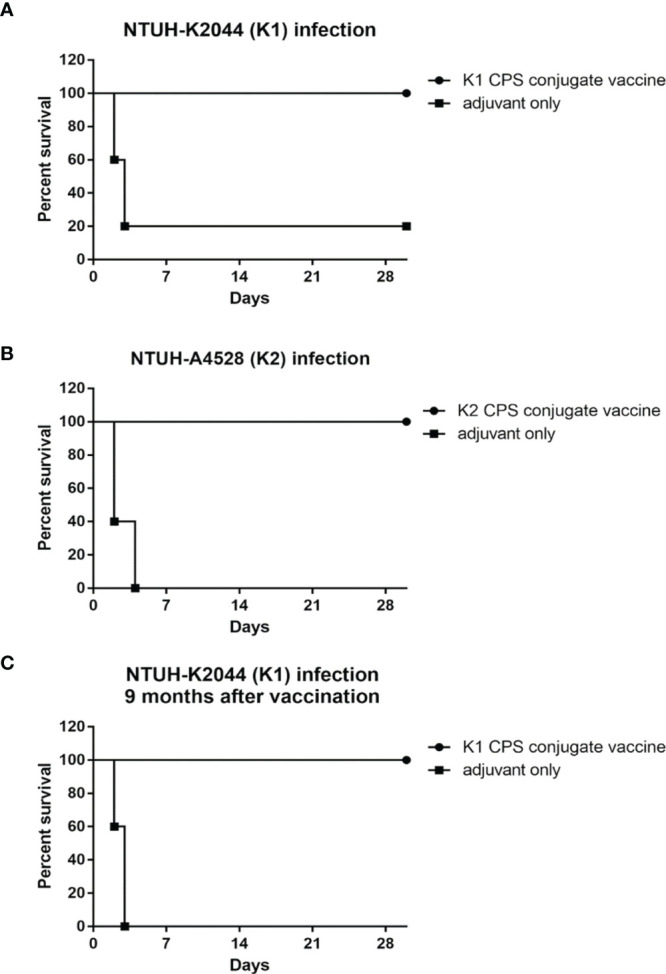
The efficacy of K1 or K2 CPS-conjugated vaccine in the mouse model. Mice (five per group) immunized with K1 or K2 CPS-conjugated vaccine were challenged by IP injections of 1×10^4^ CFU of *K. pneumoniae* NTUH-K2044 **(A)** or NTUH-A4528 **(B)** per mouse. Vaccination with K1 or K2 CPS-conjugated vaccine significantly increased the survival rate of mice that were challenged by infection of *K. pneumoniae* NTUH-K2044 (*P*=0.0144, log-rank test) or NTUH-A4528 (*P*=0.0023, log-rank test), respectively. **(C)** Mice (five per group) immunized with K1 CPS-conjugated vaccine were challenged by IP injections of 1×10^4^ CFU of *K. pneumoniae* NTUH-K2044 per mouse after nine months. And the results showed that the vaccination with K1 CPS-conjugated vaccine from 9 months ago still significantly increased the survival rate of mice that were challenged by infection of *K. pneumoniae* NTUH-K2044 (*P* = 0.0031, log-rank test). Both groups were protected from pyogenic liver abscess and mortality.

The immunized mice were challenged with *K. pneumoniae* to evaluate its *in vivo* protective effect. Experimental mice were inoculated with K1 or K2 CPS-conjugated vaccine by IM injection once a week for 3 weeks; the control animals were administered with adjuvant only on the same schedule. One week after the third vaccination, the mice were infected with 1×10^4^ CFU *K. pneumoniae* NTUH-K2044 (**Figure 7A**) or NTUH-A4528 per mouse by IP injection (**Figure 7B**). Observation over the 30 days after the *K. pneumoniae* challenge revealed significant higher survival rate of the mice that received the K1 or K2 CPS-conjugated vaccine than that of the mice that received adjuvant only.

Mice received K1 CPS-conjugated vaccine were challenged with *K. pneumoniae* NTUH-K2044 at nine months after the third vaccination (**Figure 7C**). The results indicated that the vaccine still has effective protection nine months after the inoculation. Both groups were protected from pyogenic liver abscess and mortality.

### Efficacy of K1 and K2 CPS-Conjugated Divalent Vaccines

**Figure 8:**
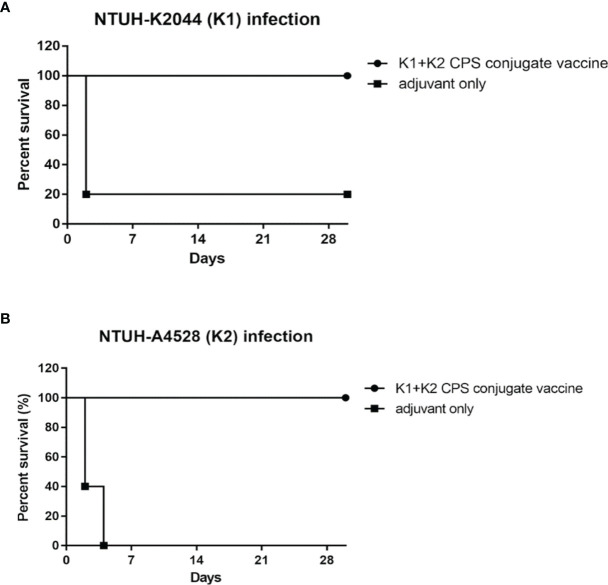
The efficacy of the K1 and K2 CPS-conjugated divalent vaccine in the mouse model. Mice (five per group) immunized with K1 and K2 CPS-conjugated divalent vaccine were challenged by IP injection of 1×10^4^ CFU of *K. pneumoniae* NTUH-K2044 **(A)** or NTUH-A4528 **(B)** per mouse. Vaccination with K1 and K2 CPS-conjugated divalent vaccine significantly increased the survival rate of mice that were challenged by infection of *K. pneumoniae* NTUH-K2044 (*P* = 0.0143, log-rank test) or NTUH-A4528 (*P* = 0.0023, log-rank test), respectively.

K1 and K2 are the prevalent capsular types of *K. pneumoniae* strains and cause invasive infections. Therefore, the protection efficacy of a divalent vaccine (a mixture of equal amounts of K1 and K2 CPS-conjugated vaccines) was further examined (**Figures 8A, B**). Mice infected with 1 × 10^4^ CFU of NTUH-K2044 or NTUH-A4528 by IP injection resulted in 80% and 100% death among adjuvant-immunized (control) mice, respectively. In contrast, no mortality was observed among divalent-vaccinated mice after IP injection. Thus, we can conclude that the divalent vaccine effectively protected mice from K1 or K2 *K. pneumoniae* infection.

## Discussion

Due to antibiotic resistance, the threat of bacterial infection to humans is increasing day by day. Glycoconjugate vaccines are widely considered to be one of the most effective strategies for preventing bacterial infections (29). State-of-the-art technologies for the production of conjugated vaccines can be classified into 3 types (**Supplementary Figure 6**). First, vaccines are prepared using purified exopolysaccharides from bacteria. This method has achieved considerable success, and most of the commercially available bacterial glycoconjugate vaccines are currently prepared in this way (**Supplementary Figure 6A1**). However, this method uses acids or bases to degrade the polysaccharides into oligosaccharides for subsequent carrier protein conjugation. If acid- or base- sensitive functional groups that are highly related to its immunogenicity exit on the exopolysaccharides, this method would not be able to produce an effective glycoconjugate vaccine. Moreover, because the fragmented oligosaccharides are in various lengths after chemical cleavage, the vaccine prepared by this method is quite complicated in heterogeneous form, which also increases the burden of CMC in the manufacture of the vaccines. Next is total synthesis by chemical methods (**Supplementary Figure 6A2**). The first marketed synthetic glycoconjugate vaccine against Hib in Cuba spurred the development of total synthesis method, and several synthetic glycoconjugate vaccines are currently in preclinical or clinical trials. Although total synthesis glycoconjugate vaccine is homogeneous, but tedious synthesis steps and high production costs are the main hurdles for this method. Finally, *in vivo* biosynthesis from *E. coli* method opened a new avenue for glycoconjugate vaccine production (**Supplementary Figure 6A3**), and several vaccines prepared by this method are also in preclinical or clinical trial phase. Because this method highly depends on the activity of PglB oligosaccharyltransferase for different *O*-antigens, glycoconjugate vaccines preparation can be speeded up if this enzyme can be evolved with high promiscuity for different *O*-antigens (30).

*K. pneumoniae* is heavily encapsulated with polysaccharides, and the *K. pneumoniae* CPS is much more mucoid and of larger molecular weight than that of other bacteria. In this study, we used phage identified capsule depolymerases to depolymerize *K. pneumoniae* CPS. The resulting oligosaccharides exhibited consistent lengths and retained important immune induction CPS modifications. Feldman et al. had used the conjugate enzyme (oligosaccharyltransferase)/target protein/polysaccharide/RmpA transcriptional activator to construct the bioconjugate vaccine against *K. pneumonia* K1 or K2 strain respectively (31). Here, we reported a successful usage of phage depolymerase for CPS depolymerization as a novel method for bacterial vaccine development (**Supplementary Figure 6B**). Notably, this work employed CPSs that were purified from mutants with deletion of *wbbO*; thus, the first dedicated enzyme in the assembly pathway was encoded the *O* antigen (32). Use of this strain background precludes potential *O*-polysaccharide contamination, a common problem in vaccine manufacturing.

Studies conducted among US Army recruits in the 1960’s demonstrated a correlation between serum bactericidal titer and protection from *N. meningitides* infection (33, 34). Thus, bactericidal antibody titer had served as a surrogate for protective immunity during the development of meningococcal vaccines. In the present study, we demonstrated safe induction of anti-capsule antibody by K1 and K2 CPS-conjugated vaccines to provide *in vivo* protection in a mouse model. Persistent (at least 9 months for K1) and high bactericidal activities of sera were observed in vaccinated mice (**Figure 4B**). If active immunization could raise the titers of protective antibodies to specific pathogens and results in protection against infection with those pathogens, immunization of injured civilians would be valuable (35). Although the association of bactericidal titer with protection from *K. pneumoniae* infection has not yet been demonstrated clinically, protection is expected in humans receiving this vaccine. Protective efficacies were both 100% for K1 and K2 vaccinations in mice by intraperitoneal challenges. However, the bactericidal titers were lower than that of K1. The protective effect of K2 vaccine could be lower than that of K1 vaccine in human, or adjust meet of K1/K2 ratio in the vaccine component may solve the difference.

Although vaccines that target bacterial capsules have achieved considerable success against infection by pathogens, the prevalence rates of capsule types are required in dealing with clinical diseases. One possible limitation for *K. pneumoniae* vaccine development is the difficulty of determining the prevalent capsular type by traditional capsular serotyping, given the poor sensitivity and cross-reactivity of the traditional assay (36). And this contributed to why no *K. pneumoniae* vaccines are licensed as of today. Recently, molecular methods, including *cps*-*wzy* PCR genotyping (37) and *wzc* sequencing (38), have been developed for capsular genotyping in *Klebsiella*. These molecular techniques have clarified the prevalence of capsule types in carbapenem-resistant *K. pneumoniae (* 39 *).* We also have completed sequence characterization of all 79 *cps* gene clusters (40); this progress is expected to remove the limitations of current genotyping methods, improve these typing methods, and provide a basis for designing vaccines to prevent *K. pneumoniae* infections. Meanwhile, according to the previous study, some CPS serotypes were poor antibody response (41), therefore, glycoconjugate vaccine might improve this weakness.

The prevalence rate of K1 and K2 capsular type is around 80-90% in community acquired pyogenic liver abscess (19), while their prevalence rate community acquired pneumonia is around 50-55% (17). Therefore, K1+K2 conjugated vaccine will be very effective in preventing community acquired pyogenic liver abscess, and also significant in prevent community acquired pneumonia. Analysis of 43 patients with hvKp liver abscess in Taiwan, it was found that a strong association between gastrointestinal colonization and liver abscess and identified carriage of hvKp isolates in healthy individuals as well (42). Other study identified carriage of hvKp in 5% of healthy Korean adults and near 6% of 1000 Asian adults surveyed (43, 44). These high rates of carriage of hvKp likely contribute to the hvKp infections in Asia. The most common capsule locus associated with hvKp is K1, K2, K5, and K57 (37, 45, 46). Further, genotype also revealed clustering of carbapenem-resistant *K. pneumoniae*, they may be the next target *K. pneumoniae* vaccine.

In conclusion, promising K1 and K2 CPS-conjugated vaccines were developed using phage depolymerases. In a mice model, the vaccines elicited immune responses with high and persistent bactericidal titers and excellent protective efficacy, demonstrating the potential for clinical application for the prevention of *K. pneumoniae* infections.

## Methods

### Phage Isolation and Sequencing

The agar overlay method was used for isolation of pure phage preparations and titer determinations of the phage. The infectivity of the phage was determined by spot test (27). Phage genomic DNA was extracted using Qiagen Lambda kit following manufacturer’s protocol with modifications as follows (27). After the phage was precipitated and lysed, phage DNA was extracted by phenol/chloroform and precipitated with ethanol. The genome sequences of the phage DNA were determined by Illumina GAII sequencing. The complete genome sequences were deposited to GenBank. The full genome of phage NTUH-K2044-K1-1 and phage 1611E-K2-1 were deposited in GenBank under accession number AB716666 (https://www.ncbi.nlm.nih.gov/search/all/?term=AB716666) and MG197810 (https://www.ncbi.nlm.nih.gov/search/all/?term=MG197810), respectively.

### Protein Expression and Purification

The recombinant His-tagged K1-ORF34 protein was expressed and purified as described in our recent study (27). The K2-ORF16 gene was amplified by PCR using primers 1611E-ORF16-F (5’-CAAACATCACGGTGACGCTAGCATGACCATTATCAAACG-3’) and 1611E-ORF16-R (5’-CTTTTAACATTTAGCACTCGAGTGTAAAATTAATAATG-3’); then, the product was digested with *Nhe*I and *Xho*I. The K2-ORF16 fragment was cloned into *Nhe*I and *Xho*I double-digested pET28c plasmid (Novagen). The resulting K2-ORF16-pET28c plasmid was transformed into *E. coli* BL21 (DE3). The recombinant His-tagged K2-ORF16 protein was expressed by inducing with 0.4 mM IPTG overnight at 16°C, and the recombinant protein was purified by nickel affinity following manufacturer’s protocol (Qiagen).

### Purification of *K. pneumoniae* K1 and K2 CPS

In order to eliminate the contamination of CPS with *O* polysaccharide, the K1 and K2 CPSs were purified from *K. pneumoniae* NTUH-K2044 Δ*wbbO* and NTUH-A4528 Δ*wbbO* mutant strains (respectively) and quantified by phenol-sulfuric acid method as previously described (47).

### Enzymatic Cleavage of CPS

The enzymatic activity of K1 lyase towards the substrate K1 CPS was evaluated by monitoring elevated absorption at 232 nm, performed at 25°C in an Ultrospec 4000 UV/Visible spectrophotometer (Amersham Pharmacia Biotech, Piscataway, NJ, USA) using a 1 mL cuvette. After K2 enzymatic reaction, the reaction supernatant was reacted with equal volume of DNSA (20 mg/mL in 0.7 M NaOH), boiled for additional 5 min, cooled on ice, and determined by monitoring the absorption at 535 nm.

The reaction for oligosaccharide production used 50 mg CPS and 100 μg enzyme in 20 mM Tris-HCl and 300 mM NaCl in pH 8 at 37°C for 4 hr. After denaturation of the enzyme, the mixture was centrifuged, and the supernatant part was separated by Biogel P6 column (1 cm D x 13 cm H) and eluted in ddH_2_O condition. The pure oligosaccharides were lyophilized for glycoconjugation.

### CPS Structure Analysis

The chemical structures of *K. pneumoniae* NTUH-K2044 (K1) and NTUH-A4528 (K2) CPS have been defined previously (26, 28). The lengths and modifications of enzyme-digested K1 and K2 CPS were determined by mass spectrometry and NMR as described in the **Supplementary Methods**.

### Conjugation of Digested K1 or K2 CPS With DT-CRM197 Carrier Protein

The digested K1 and K2 CPSs were separately conjugated with CRM197 carrier protein as described in the **Supplementary Methods**. In brief, glycosyl amines were obtained by reducing CPS *via* Kochetkov amination. A thiol linker was attached to the resulting K1/K2 digested CPS-SH. Then, the linker-modified products were reacted with CRM197-maleimid (Scheme 1). The resulting digested K1/K2 CPS-CRM197 conjugates were characterized by MALDI-TOF analysis to confirm the incorporation of carbohydrate.

### Study Approval

All animal experiments followed the guidelines in the Handbook of Laboratory Animal Care of the National Laboratory Animal Breeding and Research Center, National Science Council of Taiwan and were approved by the Institutional Animal Care and Use Committee of the National Taiwan University College of Medicine.

### Vaccination

The CPS-conjugated vaccine was diluted to 100 μg/mL in phosphate buffered saline (PBS), and the glycolipid adjuvant C34 was dissolved to 100 μg/mL in Dimethyl sulfoxide (DMSO) (48). Mice were injected intra-muscularly (IM) with 100 μL of a mixture of CPS-conjugated vaccine (2 µg K1, 2 µg K2, or 2 µg K1 plus 2 µg K2) and 2 µg adjuvant, and the booster shots were given by IM injections after one and two weeks of the inoculation. Then, sera were collected from *punctured submandibular* vein.

### Detection of Antibodies Against CPS (Dot Blot)

Purified CPSs were subjected to serial two-fold dilutions, and different amounts (ranging from 2000 ng to 7.8125 ng per spot) were transferred onto membrane by dot blotting. After incubating the dot blot with sera (1:1000 dilution) from vaccinated mice, the blots were then developed with chemiluminescent substrates per the *manufacturer’s protocol (Millipore) and exposure to X-ray films for antibody detection*.

### Serum Bactericidal Assay

Aliquots of mouse sera were inactivated by heating at 56 °C for 30 min and then subjected to two-fold serial dilution (1/2 to 1/256) with normal saline. Aliquots of 25 μL of diluted serum and 12.5 μL of fresh-cultured NTUH-K2044 or NTUH-A4528 bacterial suspension (100 CFU) were combined and incubated at 37°C for 15 minutes. After incubation, 12.5 μL of newborn rabbit complement (Pel-Freez, USA) was added to each reaction, and the mixture was incubated for 1 hour at 37 °C. Then, the reaction mixture was plated on an LB plate. After incubation of the plates overnight at 37°C, the numbers of surviving bacteria were counted. Serum bactericidal titers were defined as the reciprocal of the serum dilution that resulted in killing more than 50% of the bacteria compared to that achieved by the bacteria-complement-sera from control mice (inoculated with adjuvant only).

### Protection Assay

Vaccinated or control mice (adjuvant only) were injected intra-peritoneally (IP) with 1 × 10^4^ CFU of NTUH-K2044 or NTUH-A4528 per mouse one week after the 2^nd^ boost (5 mice per group). The mortality of the mice was tracked for 30 days. Survival was analyzed by Kaplan-Meier analysis with a log-rank test; a *P* value <0.05 was considered statistically significant. *Statistical analysis was performed using Graphpad Prism 5.*

## Data Availability Statement

The data presented in the study are deposited in the NCBI GenBank repository, accession number MG197810, AB716666.

## Ethics Statement

The animal study was reviewed and approved by 14-03-657.

## Author contributions

All authors participated in the design and interpretation of these studies. T-LL and F-LY wrote the manuscript. J-TW, S-HW, and C-YW reviewed the manuscript. All authors contributed to the article and approved the submitted version.

## Funding

This work was supported by grant funding from Ministry of Science and Technology (grants No. 103-2325-B-001-021, 104-2325-B-001-004 and 105-2325-B-001-004 to C-YW; grants No. 96-3112-B-001-020, 100-2325-B-001-021, 103-2325-B-001-020 to S-HW; grants No. 103-2325-B-002-039, 104-2325-B-002-012 and 105-2325-B-002-006 to J-TW), National Taiwan University, National Taiwan University Hospital, Academia Sinica, and the Liver Disease Prevention and Treatment Research Foundation of Taiwan.

## Conflict of Interest

The authors declare that the research was conducted in the absence of any commercial or financial relationships that could be construed as a potential conflict of interest.

The handling editor declared a past collaboration with one of the authors, CW.

## Publisher’s Note

All claims expressed in this article are solely those of the authors and do not necessarily represent those of their affiliated organizations, or those of the publisher, the editors and the reviewers. Any product that may be evaluated in this article, or claim that may be made by its manufacturer, is not guaranteed or endorsed by the publisher.
