# Supplementary material for: Development of Klebsiella pneumoniae Capsule Polysaccharide-Conjugated Vaccine Candidates Using Phage Depolymerases
Source: Front Immunol. 2022 Mar 21;13:843183. doi: 10.3389/fimmu.2022.843183 (PMC8978995; doi:10.3389/fimmu.2022.843183)

Supporting Informations

Development of *Klebsiella pneumoniae* capsule polysaccharide-conjugated vaccine candidates using phage depolymerases

Tzu-Lung Lin[†], Feng-Ling Yang[§], Chien-Tai Ren[‡], Yi-Jiun Pan[†,∞], Kuo-Shiang Liao[‡], I-Fan Tu[§], Yu-Pei Chang[§], Yang-Yu Cheng[‡], Chung-Yi Wu[‡,*], Shih-Hsiung Wu[§,*]and Jin-Town Wang[†,⊥,*]

[†]Department of Microbiology, National Taiwan University College of Medicine, Taipei, Taiwan

[§]Institute of Biological Chemistry, Academia Sinica, Taipei, Taiwan.

[‡]Genomics Research Center, Academia Sinica, Taipei, Taiwan.

[∞]Department of Microbiology, School of Medicine, China Medical University, Taichung, Taiwan.

[⊥]Department of Internal Medicine, National Taiwan University Hospital, Taipei, Taiwan

Purification of *K. pneumoniae* CPS

The bacteria were cultured on Luria agar plate over 12 hours at 37 ^{o}C. The bacteria lawn was scrapped, collected, put in sterilized water (w/v = 1/10), and heated at 100 ^{o}C for 10 minutes. After cooling, the bacterial solution was centrifuged at 15,000 x *g* for 20 minutes. The

supernatant (volume) was mixed with ice-cold acetone (4 x volumes) for polysaccharide precipitation. After centrifugation at 12,000 x *g* for 20 minutes, crude CPS was present in the pellet, which was suspended in sterilized water and lyophilized. The crude CPS powder was digested by ribonuclease (Roche) and deoxyribonuclease I (Roche) for 24 hours at 37 °C and then with protease K in 10 mM Tris-HCl, pH 7.4 for further 6 to 8 hours. After denaturation at 100 ^{o}C for 10 minutes, the supernatant was then dialyzed extensively against water using an 8-10 kDa cutoff membrane and lyophilized. The partial purified CPS was further purified on a TSK HW-65F column [1.6 cm (diameter) x 90 cm (height)] by eluting with 0.1% sodium azide in filtered H_2O. Fractions containing carbohydrates were detected by phenol-sulfuric acid method and dialyzed against H_2O (MW cutoff: 1 kDa) then concentrated by lyophilization.

CPS structure analysis

Capillary electrophoresis: Capillary electrophoresis was performed on a Beckman P/ACE MDQ Capillary Electrophoresis system equipped with a UV detector set at 230 nm. Separation and analysis were performed by an electrokinetic chromatography coated fused-silica capillary tube (77 µm ID, 65 cm total length, and 50 cm from the injection point to the detector) at 25 ^{o}C. The operating buffer, which is consisted of sodium phosphate buffer (50 mM, pH 9.0), was degassed by vacuum filtration through a 0.2 µm membrane filter and shaken in an ultrasonic bath. Before each run, the capillary tube was washed with 0.1 M NaOH for 5 minutes and

double-distilled water for 5 minutes then conditioned with the operating buffer for 5 minutes. The samples to be analyzed were injected automatically, using the pressure injection mode, in which the sample was pressurized for 15 seconds. Capillary electrophoresis was performed at 20 kV (about 65 mA) using a normal polarity.

Mass spectrometry: For MALDI MS analysis, 0.5 μL of sample was mixed with 0.5 μL of matrix solution [20 mg/mL 2, 5-dihydroxybenzoic acid (DHB) in 50% ACN and 1% H_3PO_4]. Then, sample was spotted onto stainless steel plates, air-dried, and then analyzed on a 4800 MALDI TOF/TOF Analyzer (Applied Biosystems, Foster City, CA). The analysis was carried out in positive reflector mode with an acceleration voltage of 20 kV, 16% grid voltage. A typical spectrum was generated by 1,000 laser shots. The raw spectra were processed by baseline subtraction and noise removal using Data-Explorer software (Applied Biosystems). For mass spectrum analysis, measurements were performed on an Ultraflex II MALDI-TOF/TOF mass spectrometer (Bruker Daltonik GmbH, Germany). Mass spectra were obtained in the range of mass to charge ratio (m/z) from 500 to 4,000 with reflector mode. MS/MS analysis was performed in the LIFT mode. Mass spectrometry analyses were performed in GRC Mass Core Facility of Genomics Research Center, Academia Sinica, Taipei, Taiwan.

NMR spectra: The NMR spectra of small CPS fragments in D_2O were recorded; all two-dimensional NMR experiments were carried out with standard pulse sequences provided by Bruker. NMR data were processed using topspin 3.1. NMR experiments and resonance

assignments: All NMR experiments are carried out on a Bruker AVANCE 600 or AVANCE 800 NMR spectrometer (Bruker, Karlsruhe, Germany) equipped with a triple (^{1}H, ^{13}C, and ^{15}N) resonance cryoprobe, including a shielded z-gradient. Two dimensional (2D) ^{1}HNMR, TOCSY and NOESY spectra are collected. All heteronuclear NMR experiments for recombinant protein were carried out as required. Sequence-specific assignment of the backbone atoms is achieved by independent connectivity analysis of CBCA(CO)NH, HNCACB, HNCO, HN(CA)CO, and C(CO)NH. The ^{1}H resonances are assigned using 3D HAHB(CO)NH and HCCH-TOCSY.

Conjugations of digested K1 or K2 CPS with DT-CRM197 carrier protein

General procedure for the preparation of glycosyl amines via Kochetkov amination: Ammonium carbonate (3.0 g, excess) was added to a solution of reducing sugar (20 mg) in 3.0 mL of DD water. The resulting suspension was sealed and stirred at room temperature for 7 days. The reaction mixture was freeze-dried until the dry weight of the residue stayed constant. The glycosyl amines were obtained as colorless solids and used without further purification.

General procedure for the attachment of thiol linker via glycosyl amine: DTSSP (20 mg, excess) was added to a solution of the above glycosyl amines (20 mg) in 3.0 mL of PBS buffer (pH 7.4). The reaction mixture was maintained at pH 7.4 to 7.8 by adding 1M NaOH solution and stirred at room temperature for 16 hrs. Then, DTT (20 mg, excess) was added to the above

solution, and the mixture was stirred at 40 ^{o}C for 1 to 2 hrs. The solvent was removed under reduced pressure, and the residue was purified by chromatography on a column of Sephadex LH-20 eluded with DD water to afford the desired products K1/K2 digested CPS-SH (12-15 mg, 60-75% overall) as a colorless solid.

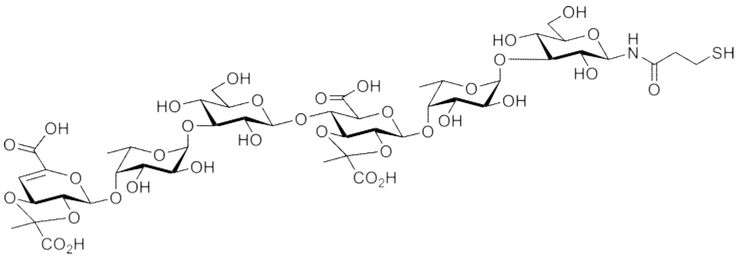

K1-digested CPS-SH: ^{1}H NMR (400 MHz, D_2O): δ= 6.20 (d, *J* = 1.9 Hz, 0.5 H), 5.73 (d, *J* = 8.4 Hz, 0.5 H), 5.39-5.30 (m, 2.4 H), 5.07-4.98 (m, 2.4 H), 4.68 (d, *J* = 7.7 Hz, 1.8 H), 4.55
(dd, *J* = 1.9, 9.4 Hz, 1 H), 4.51-4.36 (m, 4.5 H), 4.22 (m, 0.7 H), 4.15-4.04 (m, 3.4 H), 4.05-3.45 (m, 26.3 H), 2.84 (dd, *J* = 6.4, 6.8 Hz, 2 H), 2.72-2.67 (m, 1.7 H), 1.65 (s, 1.27 H), 1.62 (s, 3.7 H), 1.36-1.26 (m, 5.9 H), 1.22 (d, *J* = 6.6 Hz, 1.7 H) ppm; ^{13}C NMR (150 MHz, $CDCl_3$): δ 176.4, 175.7, 175.2, 146.8, 111.1, 109.1, 109.0, 104.8, 102.8, 102.3, 102.2, 101.6, 99.4, 99.3, 99.2, 99.1, 82.6, 81.7, 81.6, 81.5, 80.2, 79.5, 79.1, 79.0, 78.9, 78.8, 78.7, 78.1, 77.6, 76.0, 75.4, 75.3, 74.4, 73.8, 73.6, 72.3, 71.9, 69.5, 68.9, 68.8, 68.7, 68.3, 68.2, 67.8, 66.8, 66.7, 66.6, 61.0, 60.6, 39.3, 38.0, 26.7, 22.6, 19.4, 18.9, 15.2, 15.0; HRMS (ESI-TOF): m/z calcd for for $C_{45}H_{68}NO_{36}S$ 1214.3214; found 1215.3060 $(M+H+H_2O)^{+}$.

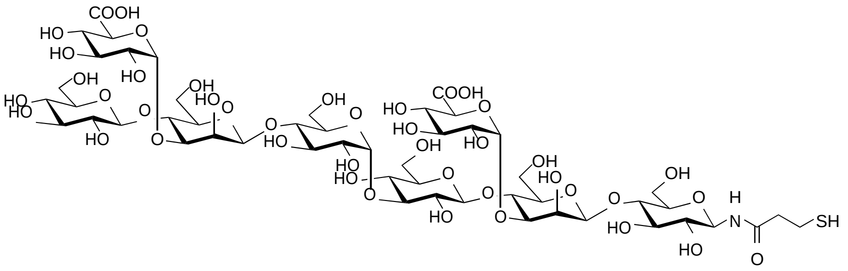

K2-digested CPS-SH: ^{1}H NMR (600 MHz, D_2O): δ = 5.38 (d, *J* = 3.8 Hz, 1 H), 5.34 (d, *J* = 4.0 Hz, 0.2 H), 5.30 (m, 2 H), 5.21 (d, *J* = 3.8 Hz, 0.9 H), 5.02 (d, *J* = 9.4 Hz, 1 H), 4.58 (d, *J* = 8.0 Hz, 0.2 H), 4.54 (d, *J* = 8.0 Hz, 0.8 H), 4.34-4.28 (m, 2.3 H), 4.25-4.16 (m, 2.6 H), 4.14-3.43 (m, 42 H), 3.38 (t, *J* = 8.7 Hz, 1 H), 2.81 (dd, *J* = 6.6, 6.8 Hz, 2 H), 2.66 (m, 2H) ppm; ^{13}C NMR (150 MHz, $CDCl_3$): δ 176.5, 176.4, 175.7, 102.4, 101.6, 100.6, 99.9, 99.7, 99.1, 99.0, 82.9, 82.4, 81.1, 81.0, 80.9, 80.7, 79.4, 78.6, 77.3, 76.3, 76.1, 75.4, 73.5, 73.0, 72.6, 72.2, 72.0, 71.8, 71.6, 71.5, 71.4, 71.3, 71.2, 70.8, 70.6, 70.3, 70.2, 70.0, 69.8, 66.0, 65.9, 65.4, 61.0, 60.9, 60.4, 59.9, 59.7, 39.3, 36.1, 19.4; HRMS (ESI-TOF): m/z calcd for $C_{51}H_{83}NO_{43}SNa$ 1452.3957; found 1452.3964 $(M+Na)^+$; calcd for $C_{51}H_{83}NO_{43}S$ 1429.4060; found 1430.4155 $(M+H)^+$.

The synthesis of *CRM197-maleimid*: After the salt of commercial *CRM197* (1.0 mg) was removed via alternate dissolving in water and dialyzing (Amicon Ultra-0.5, 10 kDa,), the residue was dissolved in PBS buffer (pH 6.5, 1.0 mL) and transferred into a sample vial. Sulfo-EMCS (1.0 mg, 8.22×10^{-6} mol) was added to the solution, and then the reaction was kept stirring at room temperature for 2 hours. The mixture was purified by Amicon Ultra-0.5 (10 kDa). After using MALDI-TOF to check the molecular weight and BCA assay to calculate the amount of protein, the *CRM197-maleimid* was stored in PBS buffer (pH 7.2, 1.0 mg/mL) for next step. The amount of maleimid function groups could be calculated based on the MALDI-TOF data. For example, with the known molecular weight of *CRM197-maleimid* 61841, the numbers of maleimide function groups on *CRM197-maleimid* can be calculated.

The synthesis of K1/K2 digested CPS-CRM197 Conjugates (1~4): The *CRM197-maleimids* were dissolved in PBS buffer (pH 7.2, 1.0 mg/mL), and then different amounts of K1/K2 digested CPS-SH (5.0 mg/mL in PBS buffer, pH 7.2) were added into the solution. The mixtures were stirred at room temperature for 2 hours. The K1/K2 digested CPS-CRM197

conjugates were purified by Amicon Ultra-0.5 (10 kDa) to remove the nonreactive K1/K2 digested CPS-SH and sodium phosphate salt via dialysis. The obtained K1/K2 digested CPS-CRM197 conjugates were characterized by MALDI-TOF analysis to determine the carbohydrate incorporation rate. The nonreactive K1/K2 digested CPS-SH could be recovered after reacting with DTT and purifying by LH-20 column chromatography. By changing the amount of K1/K2 digested CPS-SH used, we can conjugate different epitope ratio of K1/K2 oligosaccharides to the CRM197 carrier.

Supplementary Figure 1. The mass and capillary electrophoresis analysis of K1 CPS digested by K1-ORF34 protein. (A) Mass distribution of K1 CPS cleaved by K1-ORF34 and Biogel P6 separation. (B) Capillary electrophoresis of K1 CPS cleaved by K1-ORF34 protein. (C) MS-MS spectra of K1 CPS fragment (MW 1,145). (D) ^{1}H-NMR of K1-oligosaccharides.

A

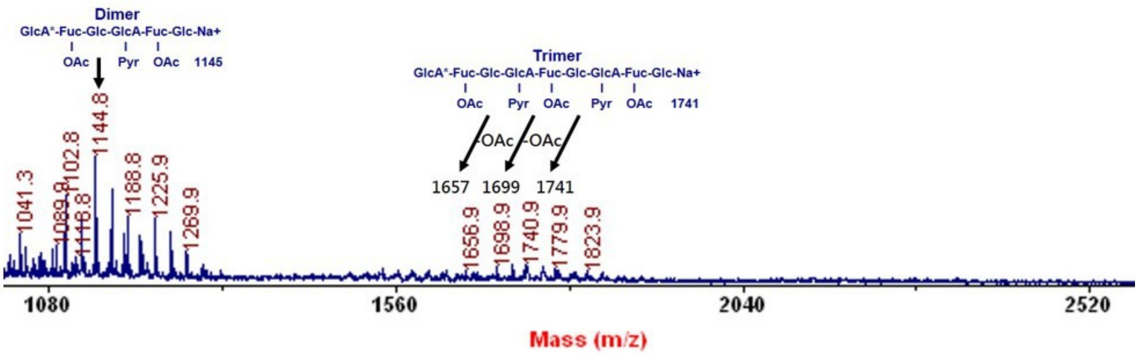

B

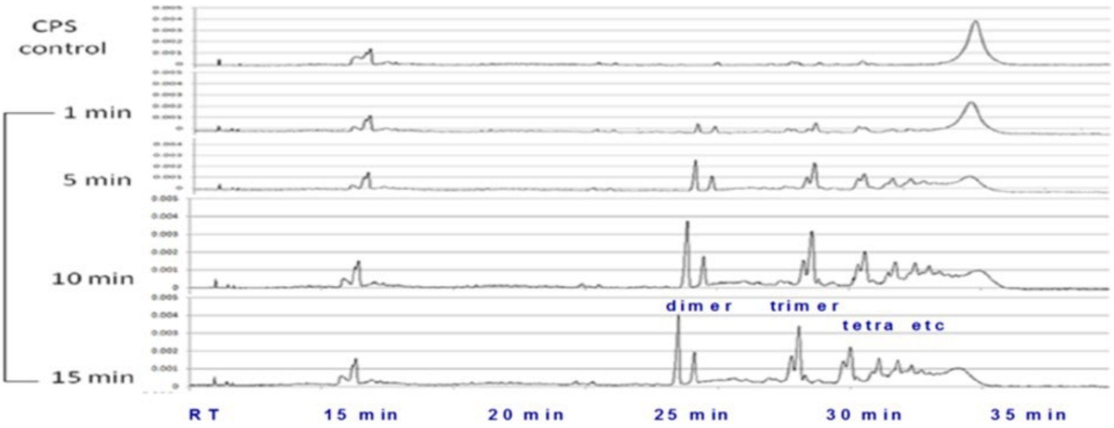

C

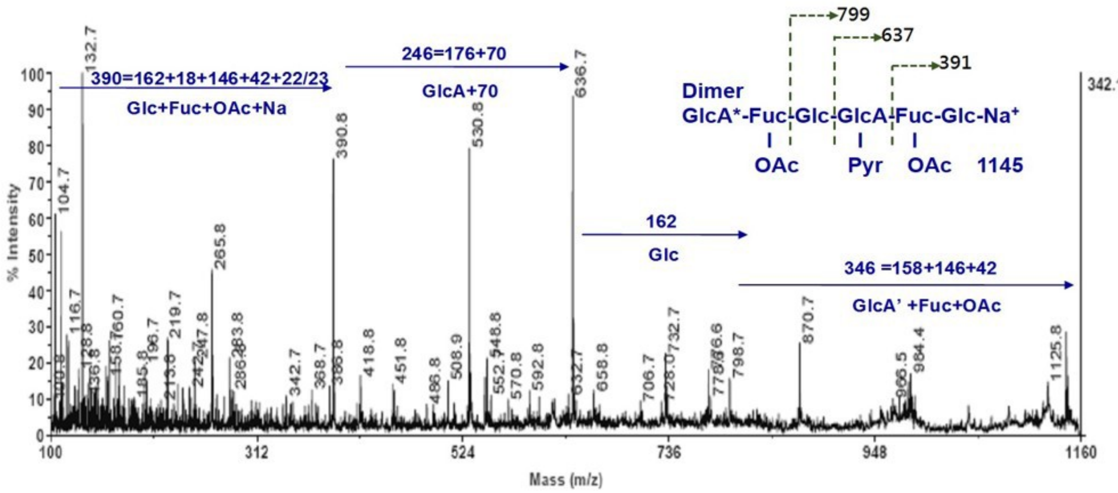
390=162+18+146+42+22/23
Glc+Fuc+OAc+Na
246=176+70
GlcA+70
162
Glc
346 =158+146+42
GlcA' +Fuc+OAc
Dimer
GlcA*-Fuc-Glc-GlcA-Fuc-Glc-Na+
OAc Pyr OAc 1145
799
637
391
342.1
% Intensity
Mass (m/z)

D

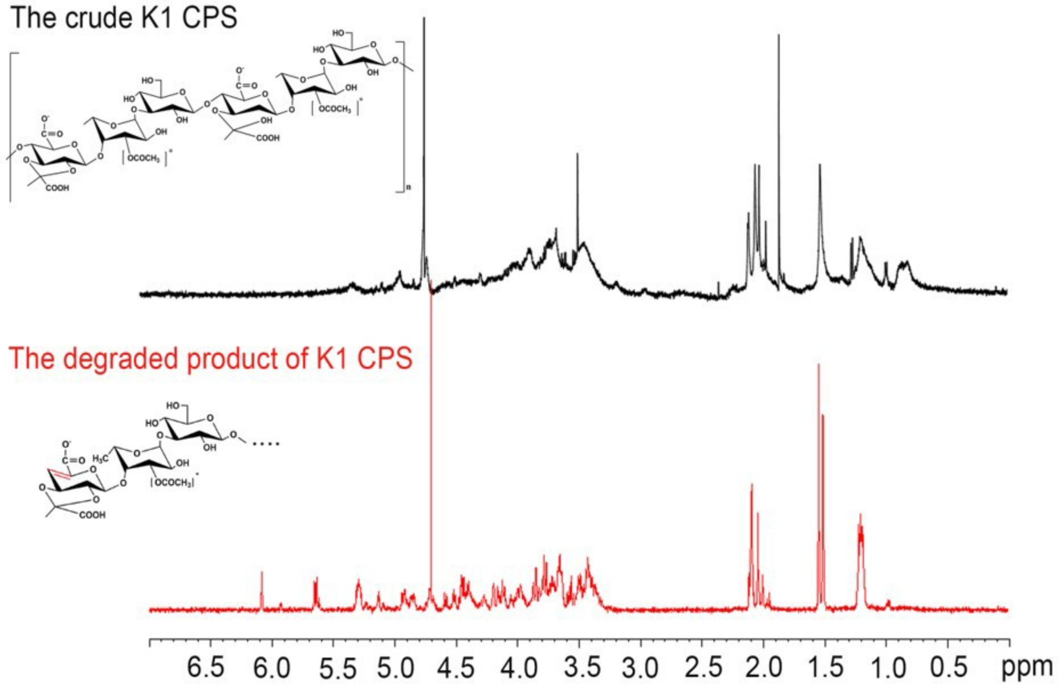
The crude K1 CPS
The degraded product of K1 CPS
ppm

Supplementary Figure 2. The structure analysis of K2 CPS digested by K2-ORF16 protein. (A) The MALDI-TOF spectra of K2 CPS digested by K2-ORF16 protein. (B) ESI-MS/MS analysis of m/z at 703 and (C) at 1365. (D) ^{13}C NMR spectrum (100 MHz) of K2 oligosaccharide (D_2O, 323 K). (E) HSQC spectrum of K2 oligosaccharide. (F) Structure of K2 oligosaccharide.

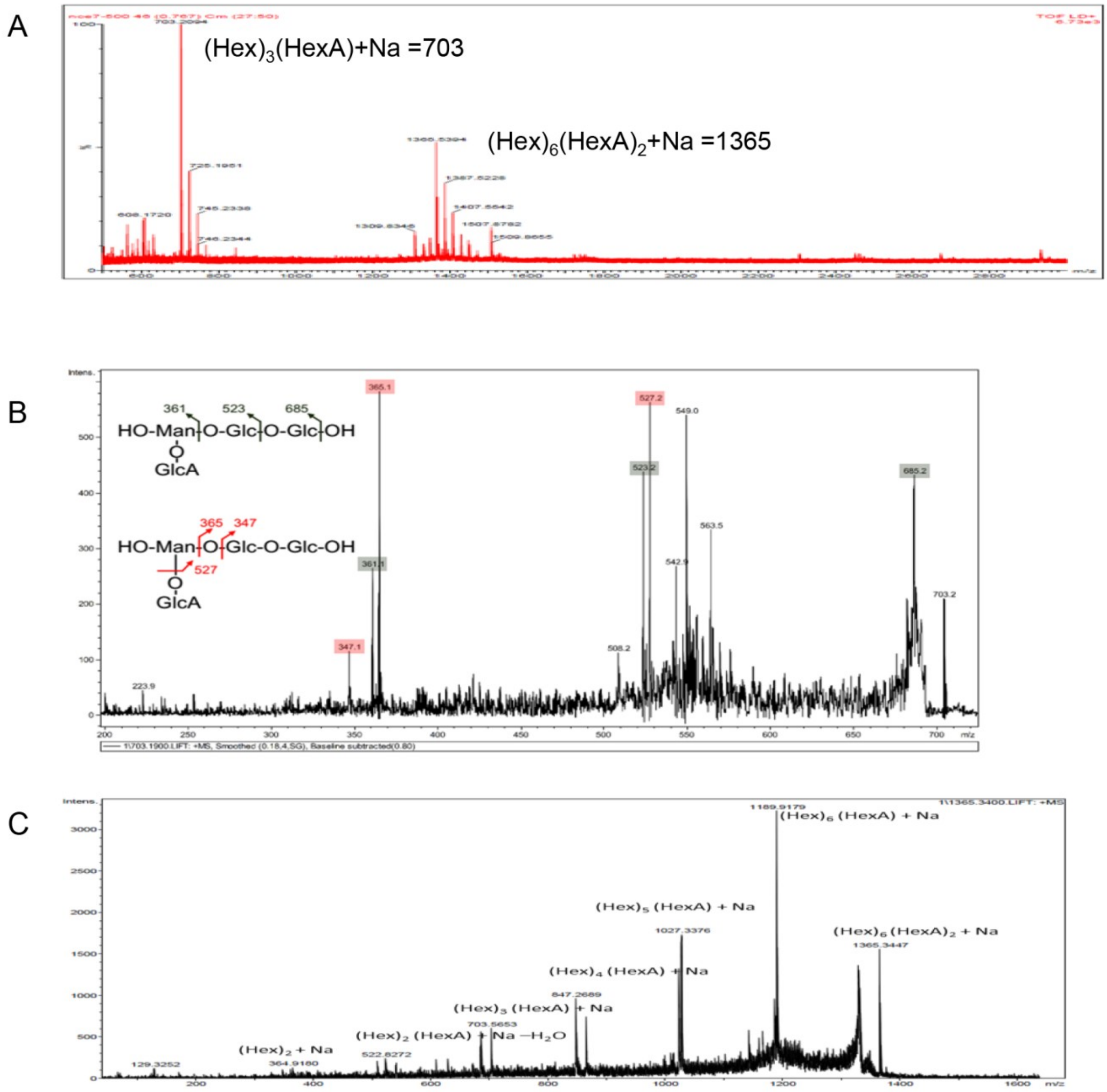

D

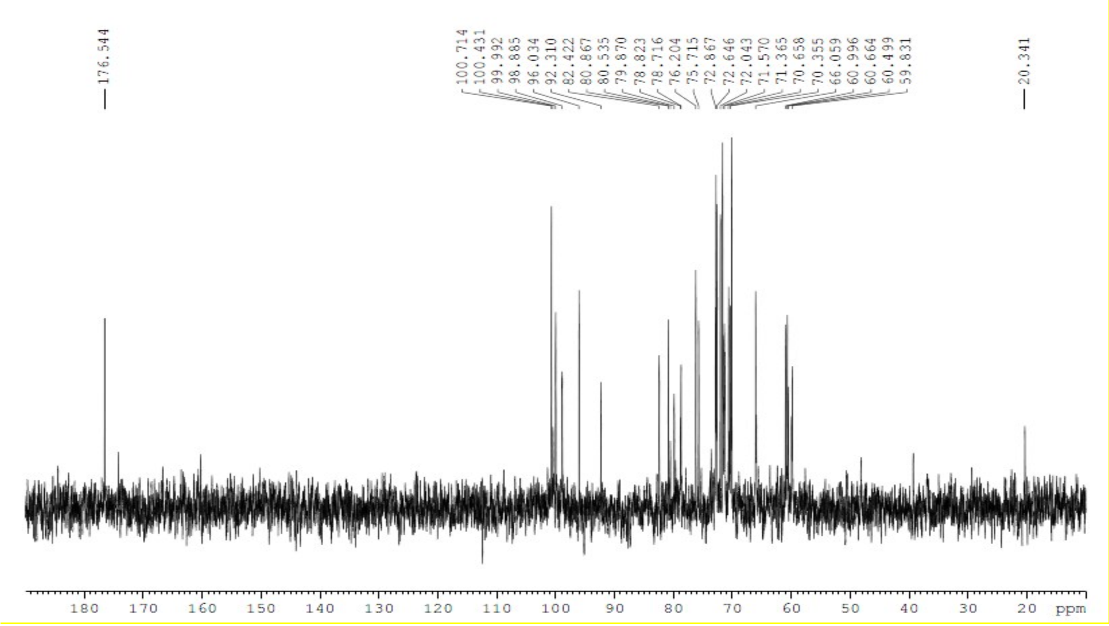
176.544
100.714
100.431
99.992
98.885
96.034
92.310
82.422
80.867
80.535
79.870
78.823
78.716
76.204
75.715
72.867
72.646
72.043
71.570
71.365
70.658
70.355
66.059
60.996
60.664
60.499
59.831
20.341
ppm

E

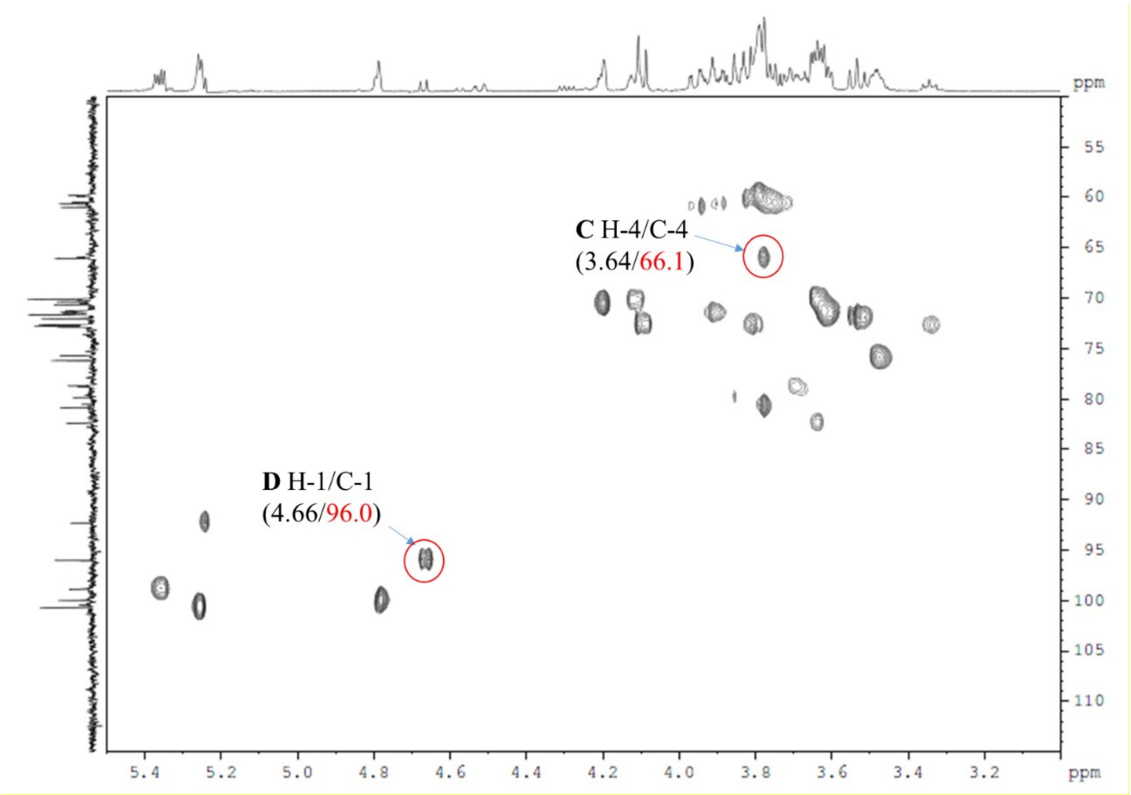
C H-4/C-4
(3.64/66.1)
D H-1/C-1
(4.66/96.0)
ppm

F

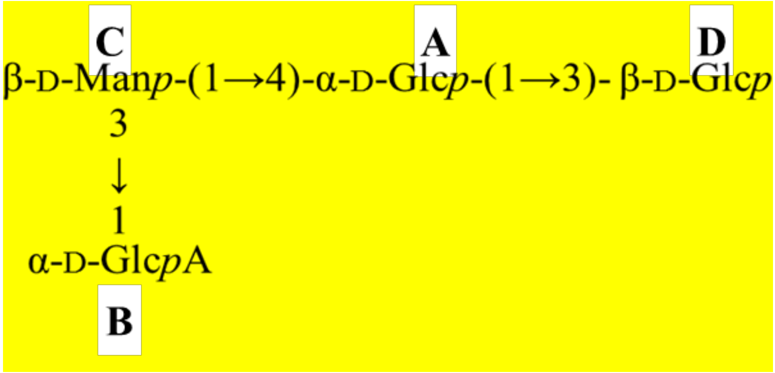

Supplementary Figure 3. The MALDI-TOF result of DT (top), linker (Sulfo-EMCS) conjugated to DT (middle), and K1 conjugated to linker of DT (down).

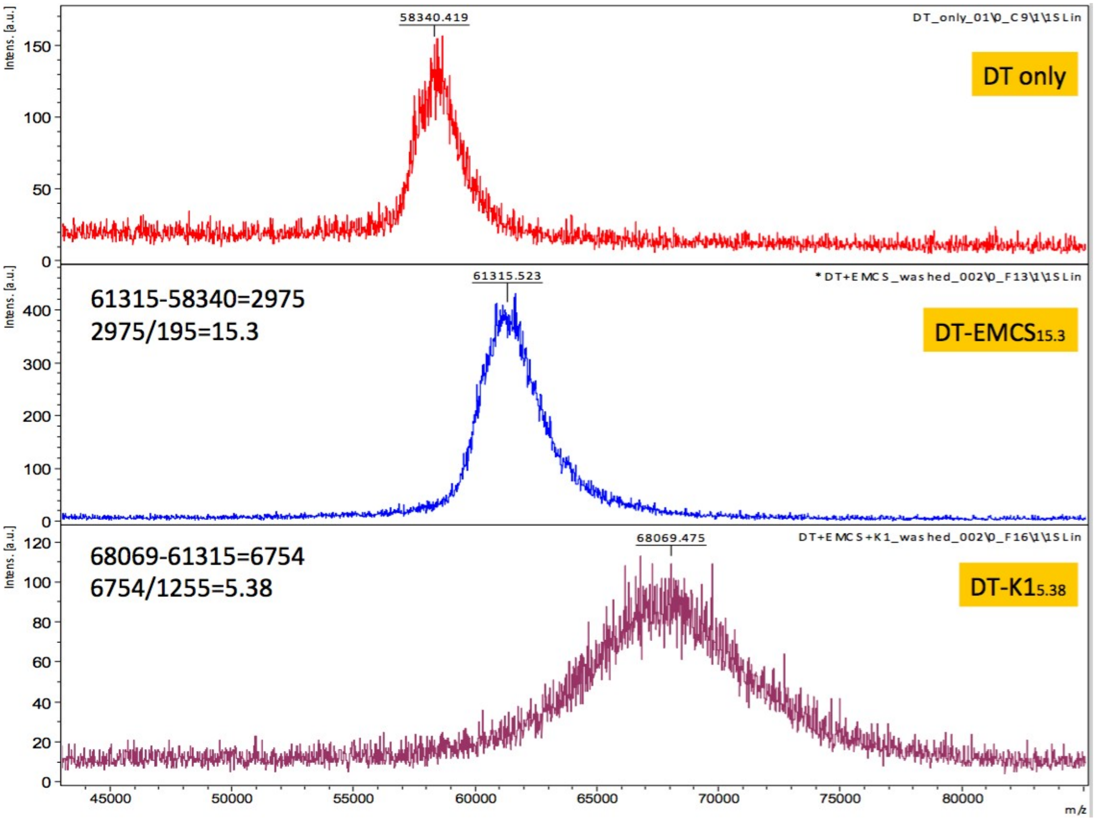

Supplementary Figure 4. The MALDI-TOF result of DT (top), linker (Sulfo-EMCS) conjugated to DT (middle), and K2 conjugated to linker of DT (down).

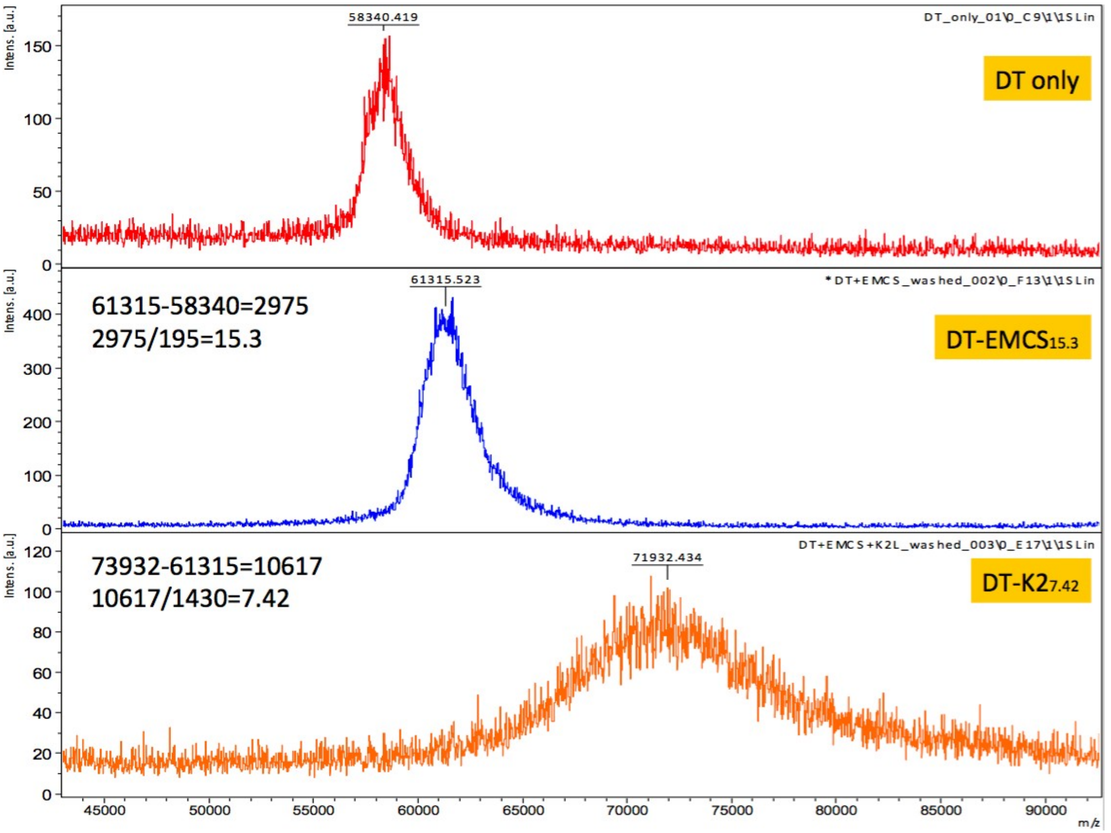

Supplementary Figure 5. The glycan array result of K1 vaccination induced IgG serogroups analysis.

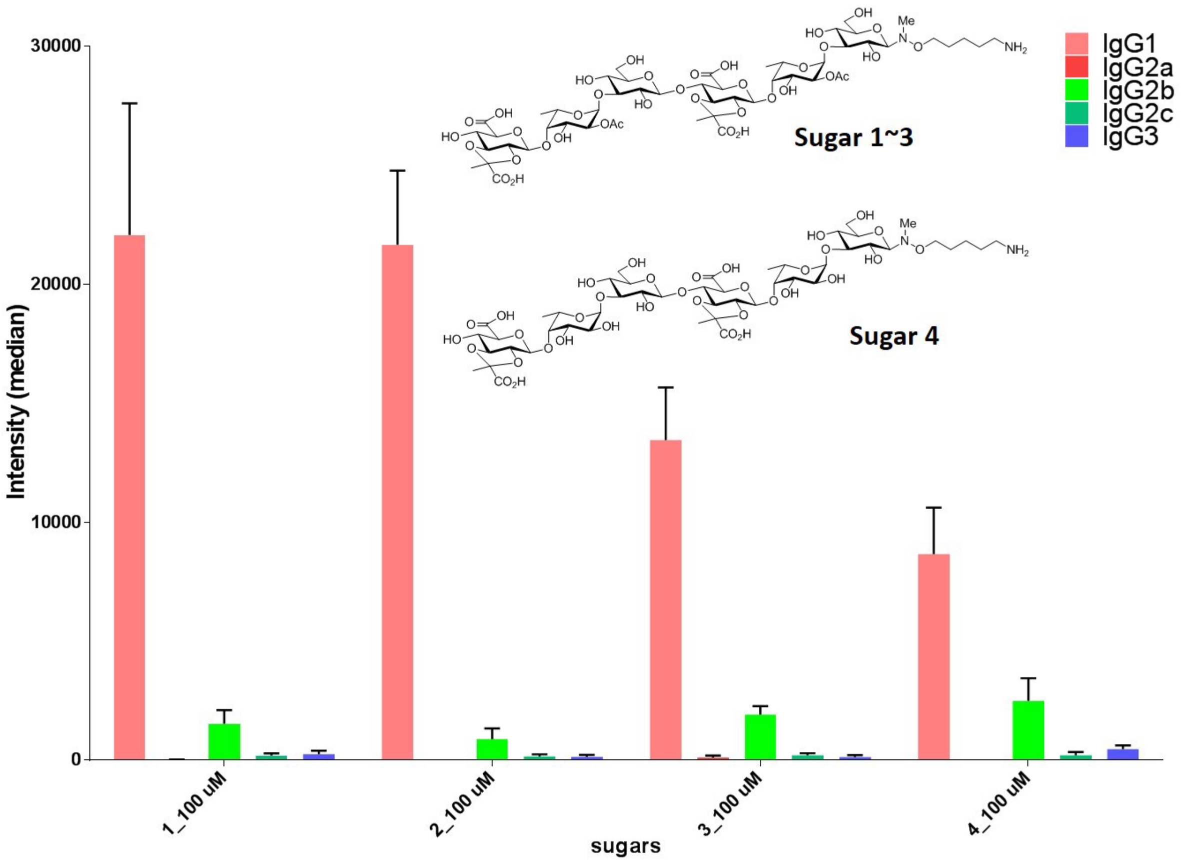

Supplementary Table I. ^{1}H and ^{13}C chemical shifts of K2 oligosaccharide

Residues & linkage	Chemical shift (*δ* ppm)					
	H-1/C-1	H-2/C-2	H-3/C-3	H-4/C-4	H-5/C-5	H-6/C-6
**A**. 4-*α*-D-Glc*p*-(1→	5.35/ 98.9	3.61/ 71.4	3.90/ 71.7	3.72/ 78.7	4.11; 70.4	3.78; 3.78/ 59.8
**B**. T-*α*-D-GlcA*p*	5.25/ 100.7	3.60/ 71.7	3.81/ 75.3	3.52/ 72.1	4.07/ 72.8	-/176.5
**C**. 3)-*β*-D-Man*p*- (1→	4.78/ 100.0	4.19/ 70.7	3.78/ 80.9	3.74/ 66.1	3.47/ 75.7	3.84; 3.94/ 60.7
**D**. T-*β*-D-Man*p*	4.66/ 96.0	3.34/ 72.7	3.64/ 82.4	3.46/ 72.1	3.46/ 76.2	3.66; 3.90/ 60.5

Supplementary Figure 6: Methods for the preparation of conjugated vaccines. (A) state-of-the-art technologies. (B) The new method reported by this study.

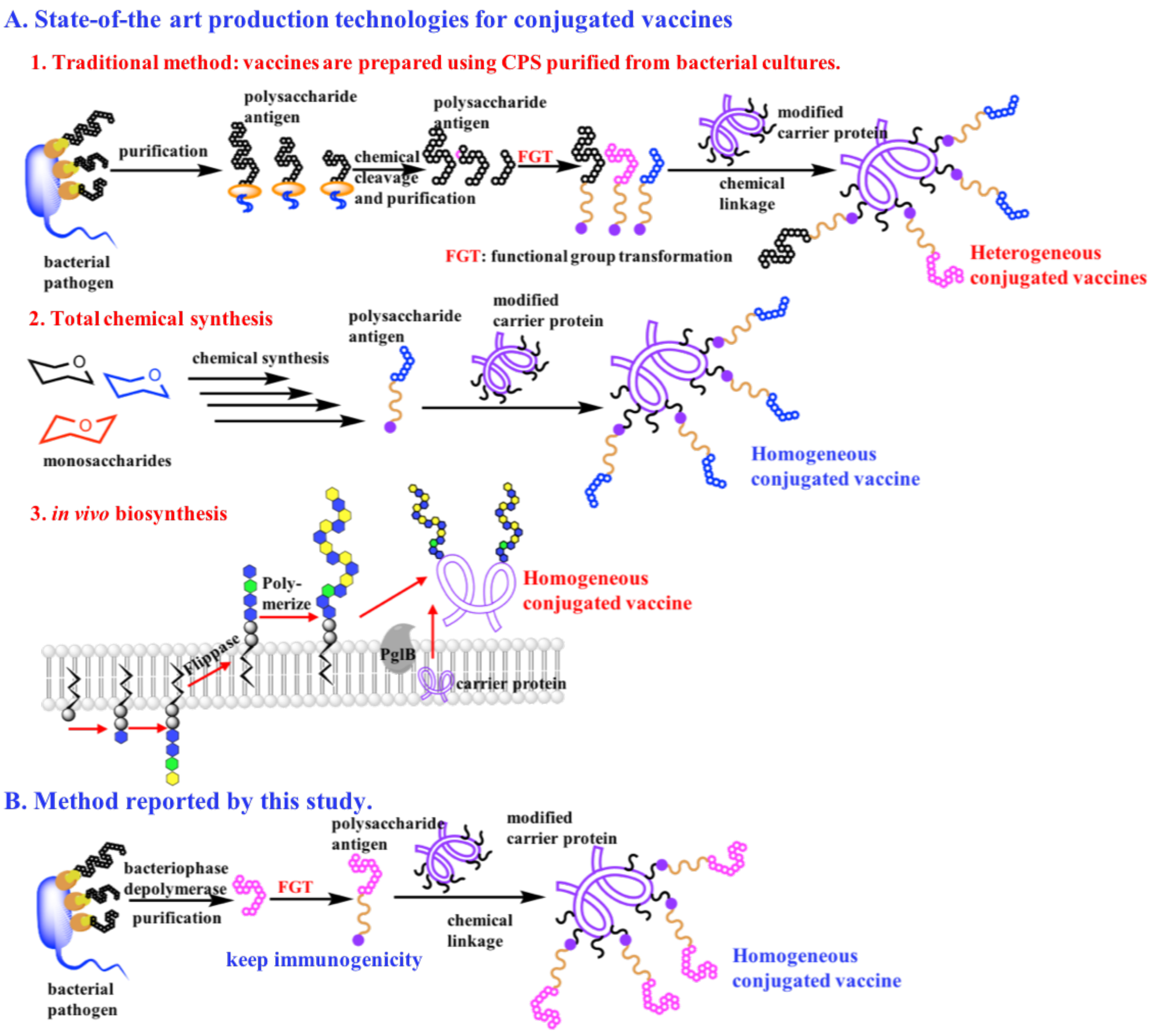

^{1}H NMR spectra of K1-digested CPS-SH

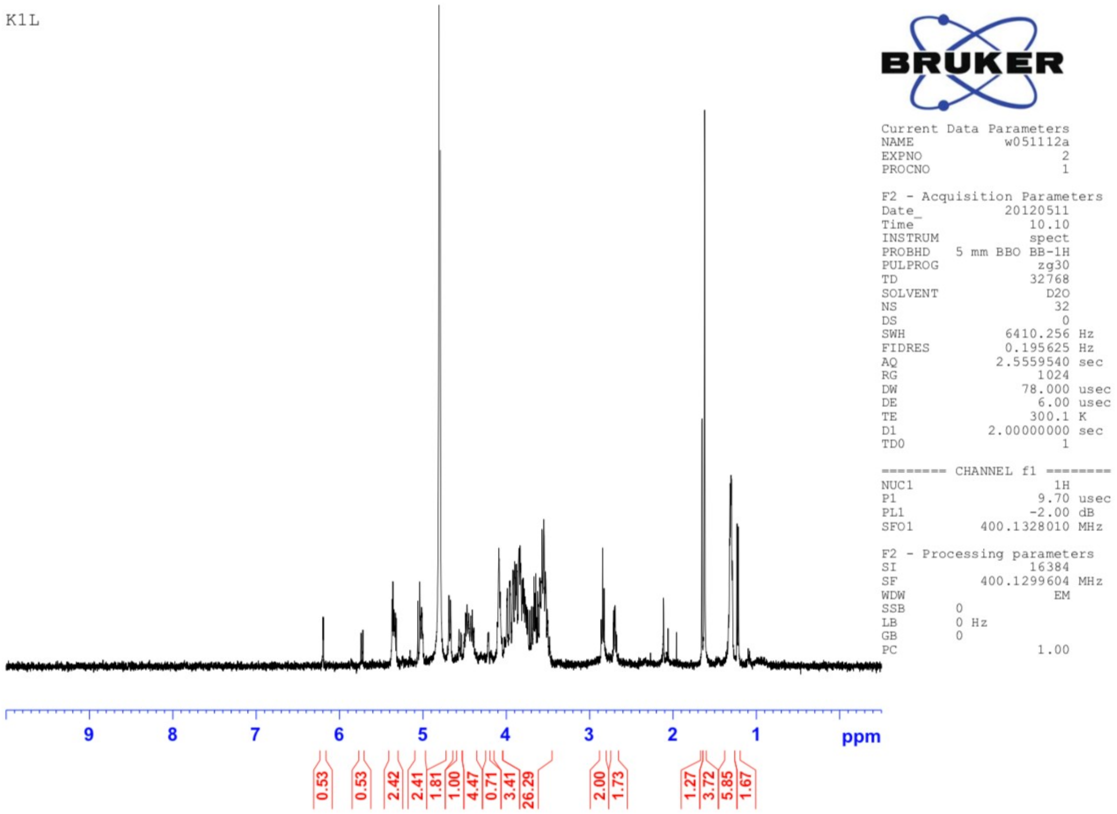

^{13}C NMR spectra of K1-digested CPS-SH

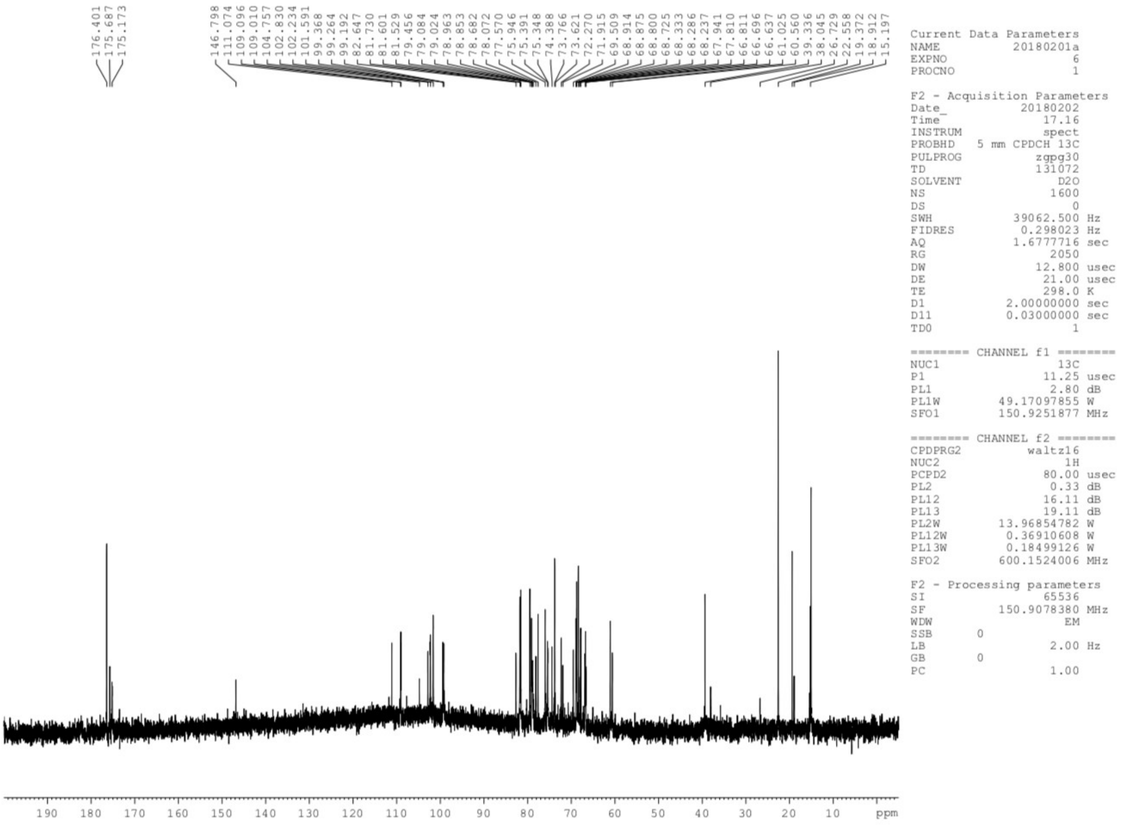

High-resolution mass spectra of K1-digested CPS-SH

Analysis Info

Analysis Name	D:\+0\Raw Data\20120308\P\abu 410 ren_62_01_6653.d	Acquisition Date	2012/3/8 下午 03:07:29
Method	ESI_Pos_500-3000	Operator	
Sample Name	abu 410 ren	Instrument	apex-Ultra
Comment			

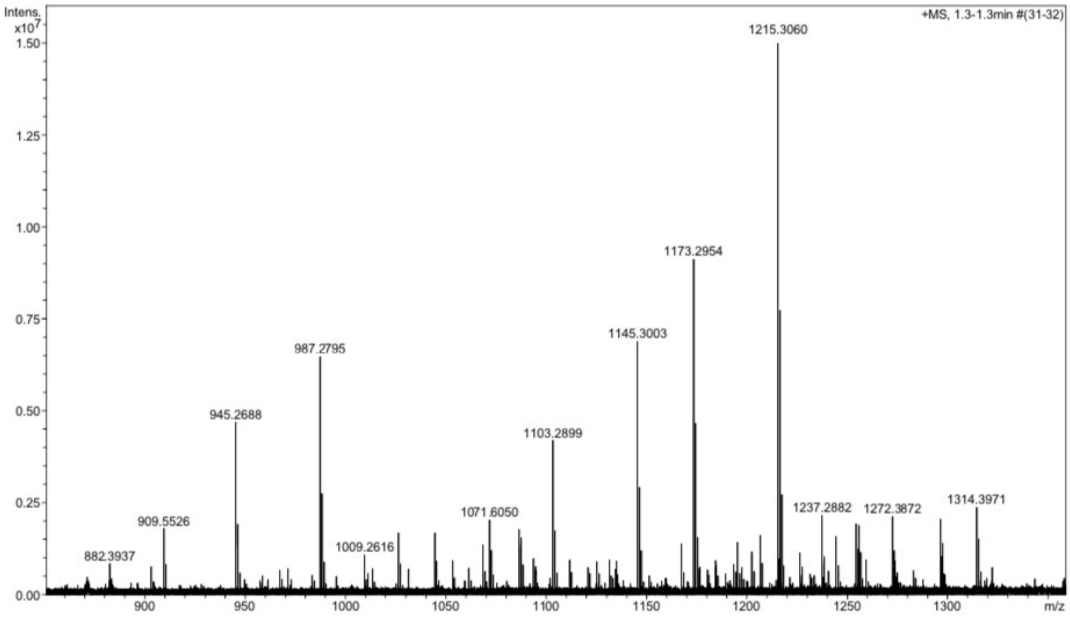

Bruker Daltonics DataAnalysis 3.4 printed: 2012/3/8 下午 04:34:57 Page 1 of 1

Generic Display Report

Analysis Info

Analysis Name	D:\+0\Raw Data\20120308\P\abu 410 ren_62_01_6653.d	Acquisition Date	2012/3/8 下午 03:07:29
Method	ESI_Pos_500-3000	Operator	
Sample Name	abu 410 ren	Instrument	apex-Ultra
Comment			

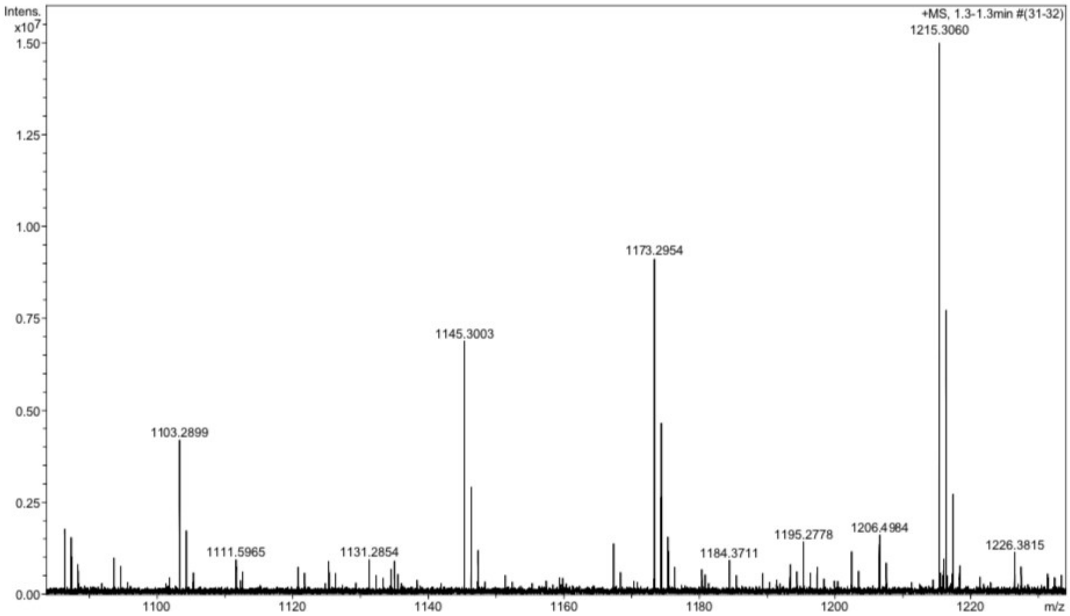

^{1}H NMR spectra of K2-digested CPS-SH

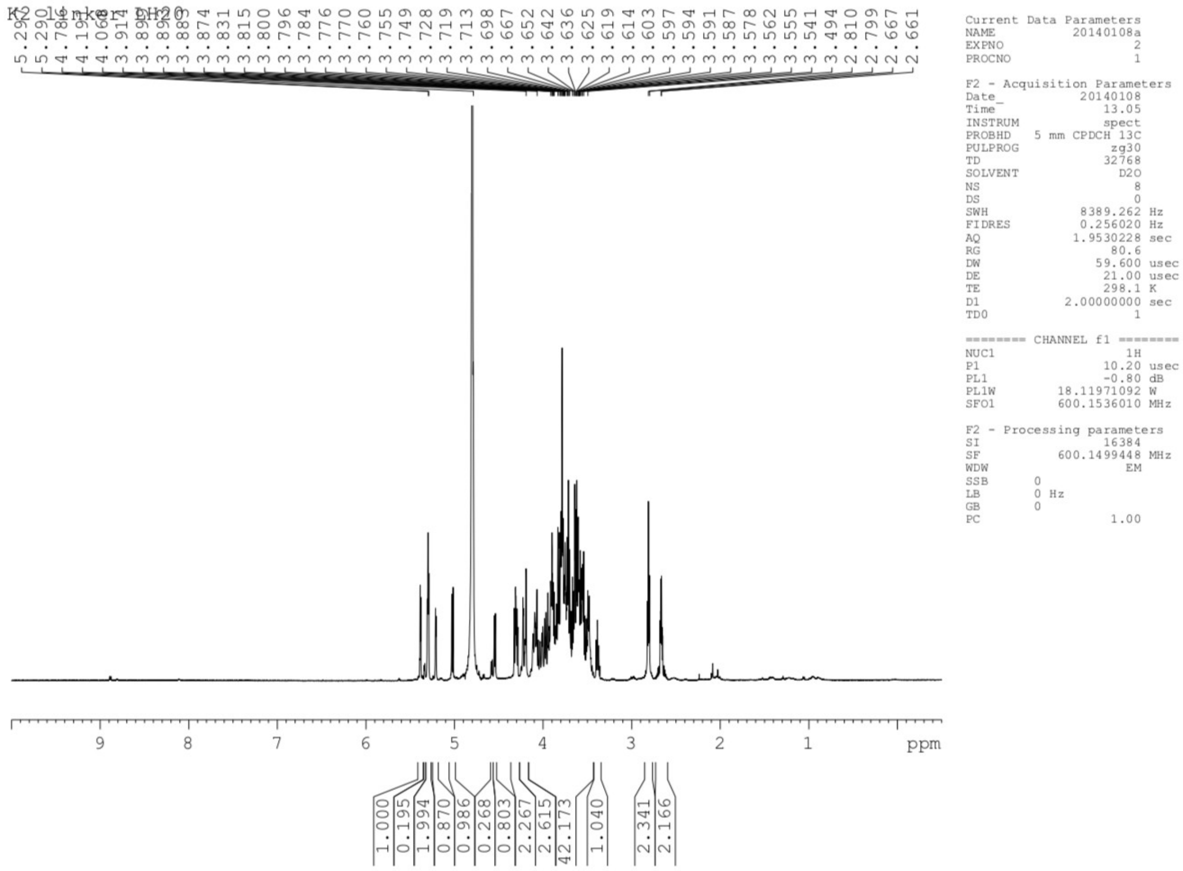

^{13}C NMR spectra of K2-digested CPS-SH

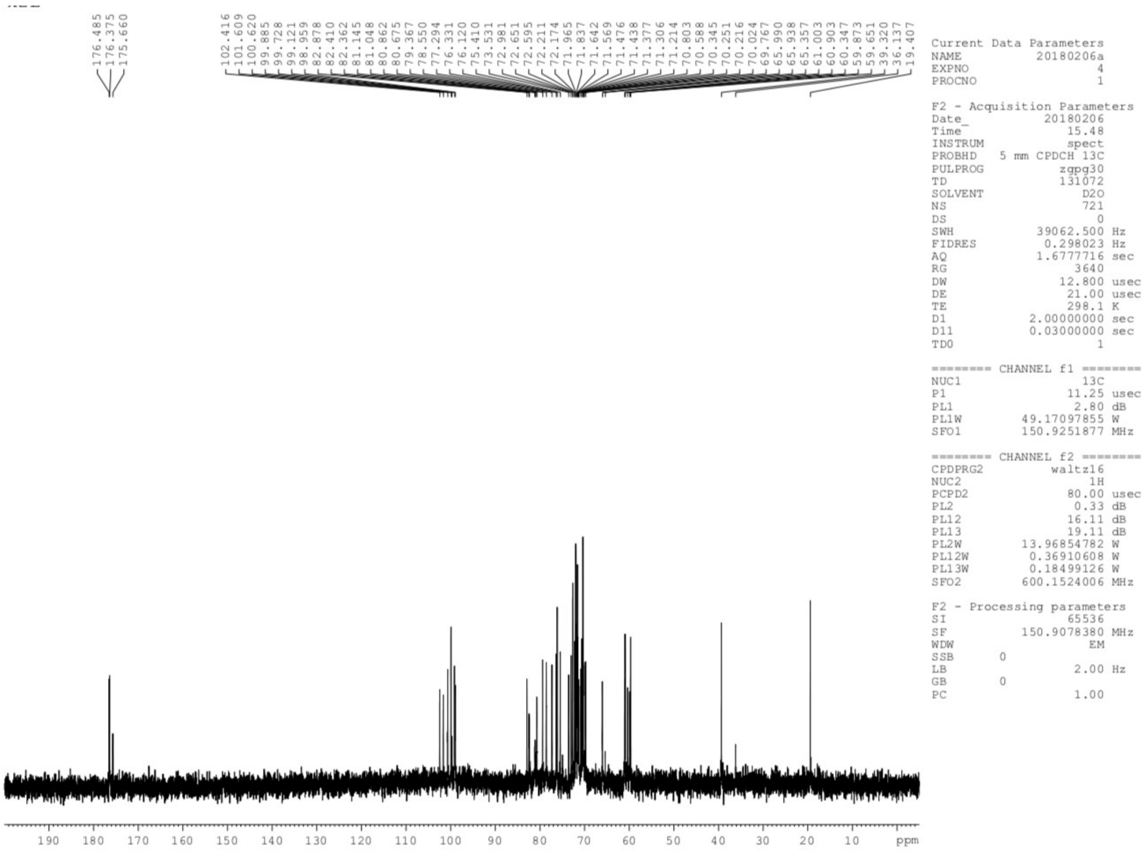

High-resolution mass spectra of K2-digested CPS-SH

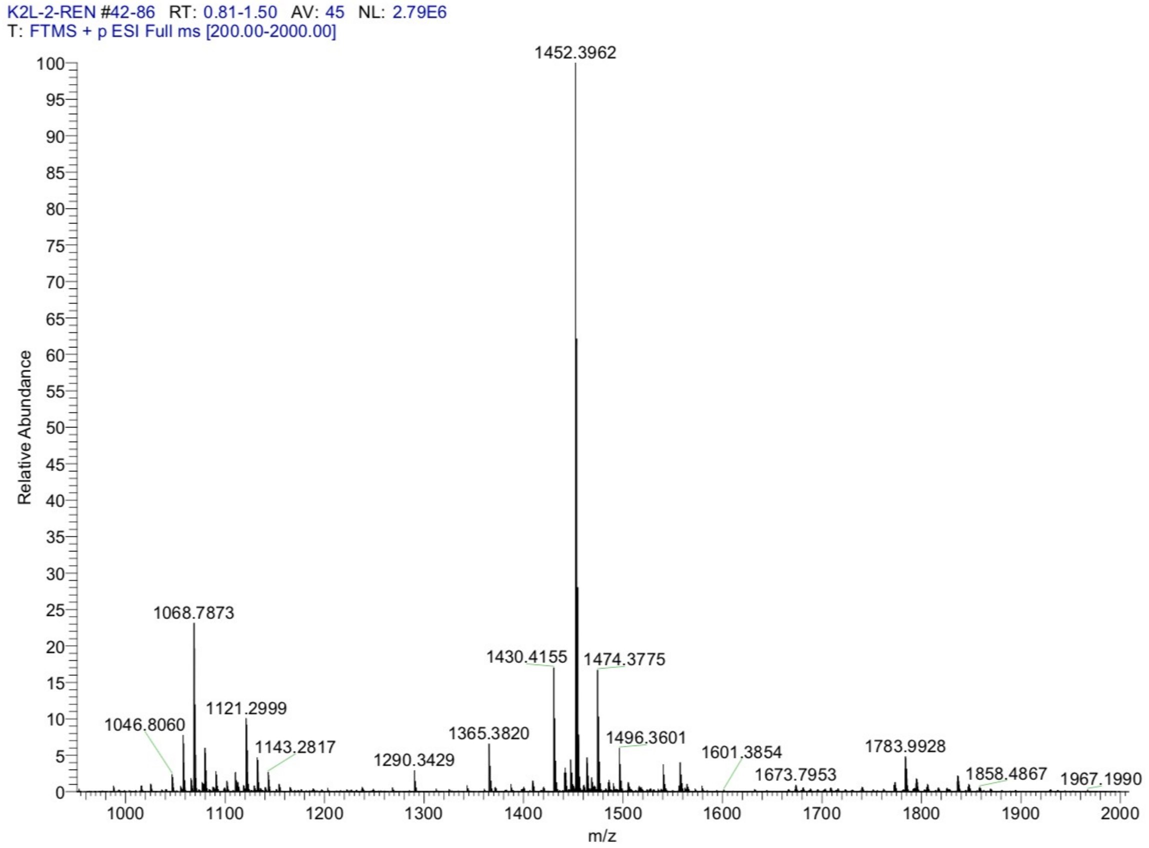

Supplement: Supplementary file 1 [file DataSheet_1.pdf]
